# Supplementary material for: Short hydrogen-bond network confined on COF surfaces enables ultrahigh proton conductivity
Source: Nat Commun. 2022 Nov 5;13:6666. doi: 10.1038/s41467-022-33868-8 (PMC9637196; doi:10.1038/s41467-022-33868-8)
Supplement: Supplementary file 1 — Supplementary Information [file 41467_2022_33868_MOESM1_ESM.pdf]

Supplementary Information for

Short hydrogen-bond network confined on COF surfaces enables ultrahigh proton conductivity

Benbing Shi[1‡], Xiao Pang[1‡], Shunning Li[3‡], Hong Wu[1,2], Jianliang Shen[1], Xiaoyao Wang[1], Chunyang Fan[1], Li Cao[1], Tianhao Zhu[1], Ming Qiu[1], Zhuoyu Yin[1], Yan Kong[1], Yiqin Liu[1], Mingzheng Zhang[3], Yawei Liu[4], Feng Pan[3*], and Zhongyi Jiang[1,2,5,6*]

1. Key Laboratory for Green Chemical Technology of Ministry of Education, School of Chemical Engineering and Technology, Tianjin University, Tianjin 300072, China.

2. Haihe Laboratory of Sustainable Chemical Transformations, 300192 Tianjin, China.

3.School of Advanced Materials, Peking University Shenzhen Graduate School, Shenzhen 518055, Guangdong, China.

4.Beijing Key Laboratory of Ionic Liquids Clean Process, CAS Key Laboratory of Green Process and Engineering, State Key Laboratory of Multiphase Complex Systems, Institute of Process Engineering, Chinese Academy of Sciences, Beijing, 100190, China.

5.Joint School of National University of Singapore and Tianjin University, International Campus of Tianjin University, Binhai New City, Fuzhou, 350207, China.

6. Zhejiang Institute of Tianjin University, Ningbo, Zhejiang 315201, China.

‡ These authors are co-first authors in this work: Benbing Shi, Xiao Pang, Shunning Li

*Correspondence to: zhyjiang@tju.edu.cn (Z.J.) and panfeng@pkusz.edu.cn (F.P.)

Supplementary Information

Figures 1 to 47

Tables 1 to 8

References (1−51)

2. Figures and Tables

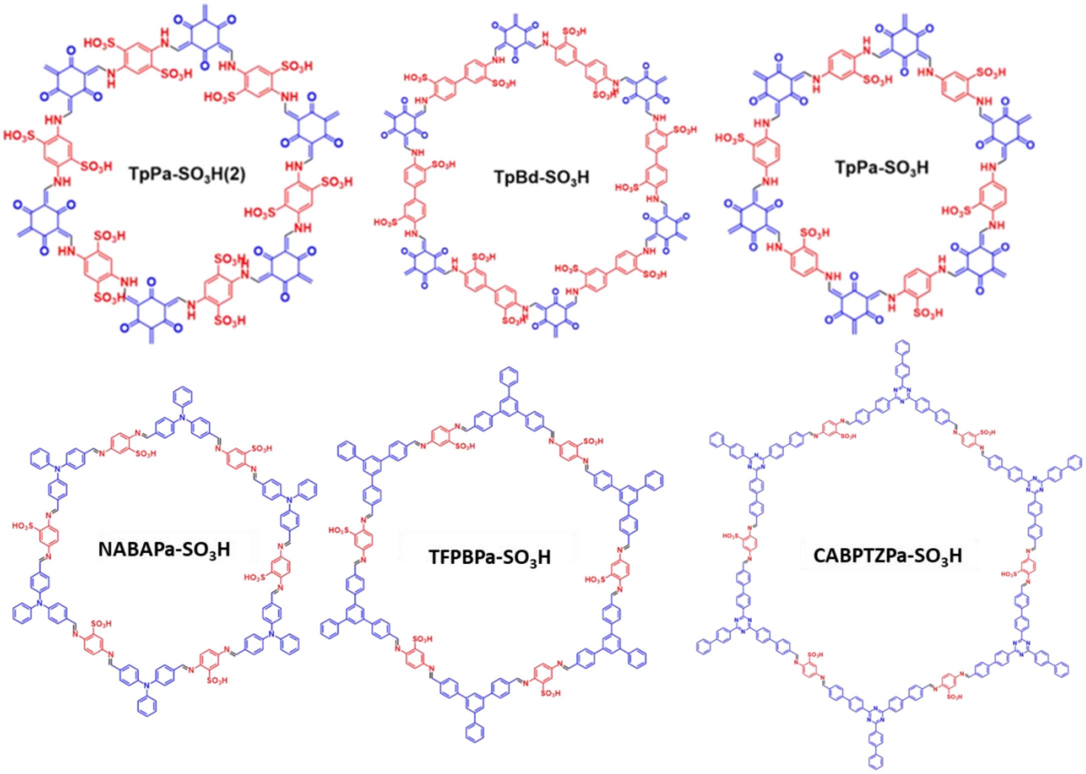

Supplementary Figure 1. The chemical structure of the iCOFs in our work.

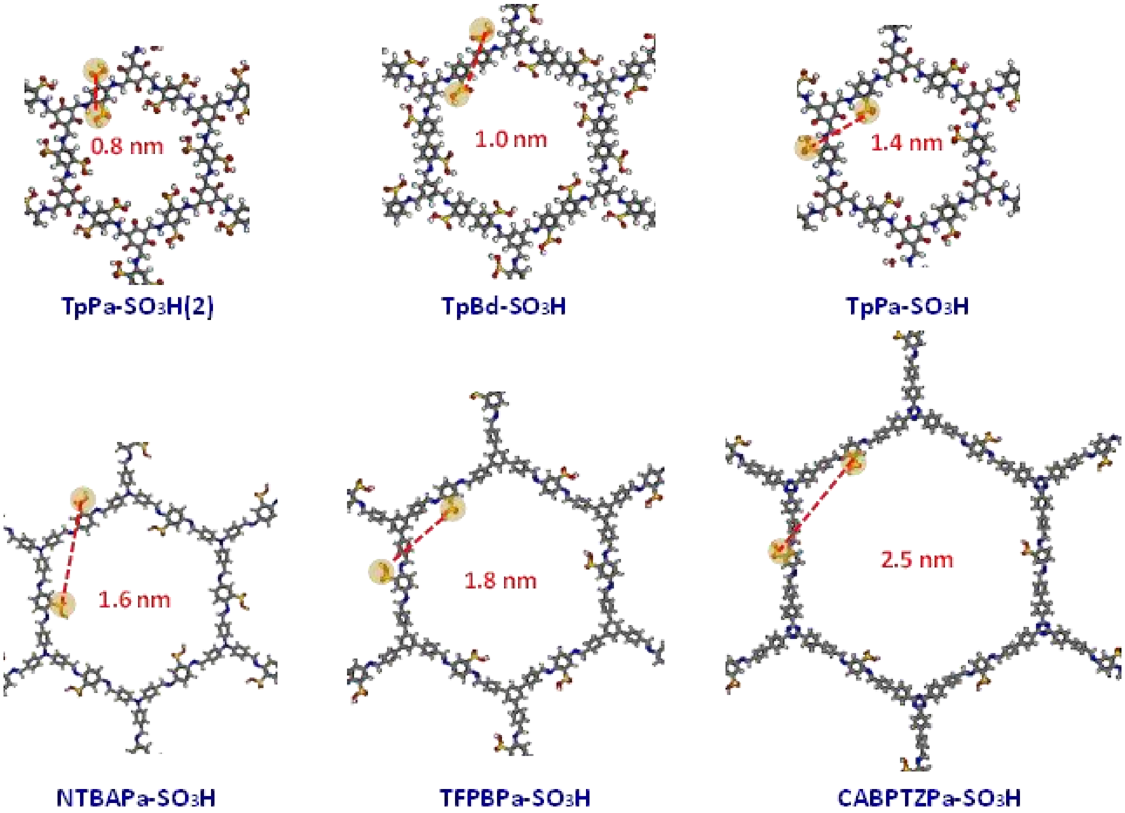

Supplementary Figure 2. The molecule stimulation of the iCOF topological structure.

Note: According to the topological structure of iCOFs, the -SO_3H group distance in TpPa-SO_3H(2), TpBd-SO_3H, TpPa-SO_3H, NTBAPa-SO_3H, TFPBPa-SO_3H, CABPTZPa-SO_3H are about 0.8 nm, 1.0 nm, 1.4 nm, 1.6 nm, 1.8 nm, 2.5 nm.

Supplementary Table 1. Eclipsed AA Unit cell parameters and fractional atomic coordinates for TpPa-SO_3H(2) calculated based on P6/M group.

Space group			P6/M	
Calculated unit cell			*a* = 23.2201 Å, *b* = 23.2201 Å, *c* = 5.5656 Å,<br>$\alpha = \beta$ =90° γ = *120*°	
atoms	Atoms number	x	y	z
H	1	0.73902	0.60932	0.5
H	2	0.36531	0.51827	0.5
H	3	0.48074	0.65595	0.5
H	4	0.54451	0.62125	0.5
H	5	-0.60932	0.1297	0.5
H	6	-0.51827	-0.15296	0.5
H	7	-0.65595	-0.17521	0.5
H	8	-0.62125	-0.07674	0.5
H	9	-0.1297	-0.73902	0.5
H	10	0.15296	-0.36531	0.5
H	11	0.17521	-0.48074	0.5
H	12	0.07674	-0.54451	0.5
H	13	-0.73902	-0.60932	0.5
H	14	-0.36531	-0.51827	0.5
H	15	-0.48074	-0.65595	0.5
H	16	-0.54451	-0.62125	0.5
H	17	0.60932	-0.1297	0.5
H	18	0.51827	0.15296	0.5
H	19	0.65595	0.17521	0.5
H	20	0.62125	0.07674	0.5
H	21	0.1297	0.73902	0.5
H	22	-0.15296	0.36531	0.5
H	23	-0.17521	0.48074	0.5
H	24	-0.07674	0.54451	0.5
C	1	0.30892	0.59719	0.5
C	2	0.3818	0.64045	0.5
C	3	0.4276	0.61957	0.5
C	4	0.45538	0.5256	0.5
C	5	0.52402	0.56695	0.5
C	6	0.56971	0.54395	0.5
C	7	-0.59719	-0.28827	0.5
C	8	-0.64045	-0.25865	0.5
C	9	-0.61957	-0.19197	0.5
C	10	-0.5256	-0.07022	0.5
C	11	-0.56695	-0.04293	0.5
C	12	-0.54395	0.02576	0.5
C	13	0.28827	-0.30892	0.5
C	14	0.25865	-0.3818	0.5
C	15	0.19197	-0.4276	0.5
C	16	0.07022	-0.45538	0.5
C	17	0.04293	-0.52402	0.5

C	18	-0.02576	-0.56971	0.5
C	19	-0.30892	-0.59719	0.5
C	20	-0.3818	-0.64045	0.5
C	21	-0.4276	-0.61957	0.5
C	22	-0.45538	-0.5256	0.5
C	23	-0.52402	-0.56695	0.5
C	24	-0.56971	-0.54395	0.5
C	25	0.59719	0.28827	0.5
C	26	0.64045	0.25865	0.5
C	27	0.61957	0.19197	0.5
C	28	0.5256	0.07022	0.5
C	29	0.56695	0.04293	0.5
C	30	0.54395	-0.02576	0.5
C	31	-0.28827	0.30892	0.5
C	32	-0.25865	0.3818	0.5
C	33	-0.19197	0.4276	0.5
C	34	-0.07022	0.45538	0.5
C	35	-0.04293	0.52402	0.5
C	36	0.02576	0.56971	0.5
N	1	0.41383	0.55465	0.5
N	2	-0.55465	-0.14082	0.5
N	3	0.14082	-0.41383	0.5
N	4	-0.41383	-0.55465	0.5
N	5	0.55465	0.14082	0.5
N	6	-0.14082	0.41383	0.5
O	1	0.67107	0.63787	0.72028
O	2	-0.63787	0.0332	0.72028
O	3	-0.0332	-0.67107	0.72028
O	4	-0.67107	-0.63787	0.72028
O	5	0.63787	-0.0332	0.72028
O	6	0.0332	0.67107	0.72028
O	7	-0.67107	-0.63787	-0.72028
O	8	0.63787	-0.0332	-0.72028
O	9	0.0332	0.67107	-0.72028
O	10	0.67107	0.63787	-0.72028
O	11	-0.63787	0.0332	-0.72028
O	12	-0.0332	-0.67107	-0.72028
O	13	0.2872	0.53751	0.5
O	14	0.69805	0.56865	0.5
O	15	-0.53751	-0.25031	0.5
O	16	-0.56865	0.1294	0.5
O	17	0.25031	-0.2872	0.5
O	18	-0.1294	-0.69805	0.5
O	19	-0.2872	-0.53751	0.5
O	20	-0.69805	-0.56865	0.5
O	21	0.53751	0.25031	0.5
O	22	0.56865	-0.1294	0.5
O	23	-0.25031	0.2872	0.5
O	24	0.1294	0.69805	0.5

S	1	0.65581	0.6032	0.5
S	2	-0.6032	0.05261	0.5
S	3	-0.05261	-0.65581	0.5
S	4	-0.65581	-0.6032	0.5
S	5	0.6032	-0.05261	0.5
S	6	0.05261	0.65581	0.5

Supplementary Table 2. Eclipsed AA Unit cell parameters and fractional atomic coordinates for TpBd-SO_3H calculated based on P6/M group.

Space group			P6/M	
Calculated unit cell			*a* = 23.1224 Å, *b* = 23.1224 Å, *c* = 5.3815 Å,<br>*α* = *β* =90° *γ = 120°*	
atoms	Atoms number	x	y	z
H	1	0.38954	0.44068	0.5
H	2	0.49151	0.673	0.5
H	3	0.5626	0.63736	0.5
H	4	0.3815	0.53197	0.5
H	5	0.52348	0.35275	0.5
H	6	0.46094	0.39092	0.5
H	7	0.64346	0.48816	0.5
H	8	0.75183	0.61356	0.5
H	9	-0.44068	-0.05114	0.5
H	10	-0.673	-0.18149	0.5
H	11	-0.63736	-0.07476	0.5
H	12	-0.53197	-0.15047	0.5
H	13	-0.35275	0.17073	0.5
H	14	-0.39092	0.07002	0.5
H	15	-0.48816	0.1553	0.5
H	16	-0.61356	0.13827	0.5
H	17	0.05114	-0.38954	0.5
H	18	0.18149	-0.49151	0.5
H	19	0.07476	-0.5626	0.5
H	20	0.15047	-0.3815	0.5
H	21	-0.17073	-0.52348	0.5
H	22	-0.07002	-0.46094	0.5
H	23	-0.1553	-0.64346	0.5
H	24	-0.13827	-0.75183	0.5
C	1	0.31605	0.59832	0.5
C	2	0.38815	0.64838	0.5
C	3	0.43891	0.63407	0.5
C	4	0.47148	0.54322	0.5
C	5	0.4442	0.47384	0.5
C	6	0.54074	0.58267	0.5
C	7	0.69437	0.40362	0.5
C	8	0.62086	0.36308	0.5
C	9	0.57734	0.38712	0.5
C	10	0.55515	0.48527	0.5
C	11	0.58356	0.55581	0.5
C	12	0.48541	0.44582	0.5
C	13	-0.59832	-0.28227	0.5
C	14	-0.64838	-0.26023	0.5
C	15	-0.63407	-0.19516	0.5
C	16	-0.54322	-0.07174	0.5

C	17	-0.47384	-0.02964	0.5
C	18	-0.58267	-0.04193	0.5
C	19	-0.40362	0.29075	0.5
C	20	-0.36308	0.25778	0.5
C	21	-0.38712	0.19022	0.5
C	22	-0.48527	0.06988	0.5
C	23	-0.55581	0.02775	0.5
C	24	-0.44582	0.03959	0.5
C	25	0.28227	-0.31605	0.5
C	26	0.26023	-0.38815	0.5
C	27	0.19516	-0.43891	0.5
C	28	0.07174	-0.47148	0.5
C	29	0.02964	-0.4442	0.5
C	30	0.04193	-0.54074	0.5
C	31	-0.29075	-0.69437	0.5
C	32	-0.25778	-0.62086	0.5
C	33	-0.19022	-0.57734	0.5
C	34	-0.06988	-0.55515	0.5
C	35	-0.02775	-0.58356	0.5
C	36	-0.03959	-0.48541	0.5
N	1	0.42879	0.57054	0.5
N	2	0.59424	0.45318	0.5
N	3	-0.57054	-0.14175	0.5
N	4	-0.45318	0.14106	0.5
N	5	0.14175	-0.42879	0.5
N	6	-0.14106	-0.59424	0.5
O	1	0.68865	0.64705	0.72692
O	2	-0.64705	0.0416	0.72692
O	3	-0.0416	-0.68865	0.72692
O	4	0.68865	0.64705	-0.72692
O	5	-0.64705	0.0416	-0.72692
O	6	-0.0416	-0.68865	-0.72692
O	7	0.30027	0.53955	0.5
O	8	0.71898	0.46395	0.5
O	9	0.70993	0.57363	0.5
O	10	-0.53955	-0.23928	0.5
O	11	-0.46395	0.25503	0.5
O	12	-0.57363	0.1363	0.5
O	13	0.23928	-0.30027	0.5
O	14	-0.25503	-0.71898	0.5
O	15	-0.1363	-0.70993	0.5
S	1	0.67088	0.6118	0.5
S	2	-0.6118	0.05908	0.5
S	3	-0.05908	-0.67088	0.5

Supplementary Table 3. Eclipsed AA Unit cell parameters and fractional atomic coordinates for TpPa-SO_3H calculated based on P6/M group.

Space group			P6/M	
Calculated unit cell			*a* = 29.0899 Å, *b* = 29.0899 Å, *c* = 5.6207 Å,<br>*α* = *β* =90° *γ* = *120*°	
atoms	Atoms number	x	y	z
H	1	0.53306	0.77713	1
H	2	-0.77713	-0.24407	1
H	3	0.24407	-0.53306	1
H	4	-0.53306	-0.77713	1
H	5	0.77713	0.24407	1
H	6	-0.24407	0.53306	1
H	7	0.30176	0.57002	0
H	8	0.45102	0.66371	0
H	9	0.49903	0.63618	0
H	10	0.55249	0.59163	0
H	11	0.40867	0.43966	0
H	12	-0.57002	-0.26826	0
H	13	-0.66371	-0.21269	0
H	14	-0.63618	-0.13715	0
H	15	-0.59163	-0.03914	0
H	16	-0.43966	-0.03099	0
H	17	0.26826	-0.30176	0
H	18	0.21269	-0.45102	0
H	19	0.13715	-0.49903	0
H	20	0.03914	-0.55249	0
H	21	0.03099	-0.40867	0
H	22	-0.30176	-0.57002	0
H	23	-0.45102	-0.66371	0
H	24	-0.49903	-0.63618	0
H	25	-0.55249	-0.59163	0
H	26	-0.40867	-0.43966	0
H	27	0.57002	0.26826	0
H	28	0.66371	0.21269	0
H	29	0.63618	0.13715	0
H	30	0.59163	0.03914	0
H	31	0.43966	0.03099	0
H	32	-0.26826	0.30176	0
H	33	-0.21269	0.45102	0
H	34	-0.13715	0.49903	0
H	35	-0.03914	0.55249	0
H	36	-0.03099	0.40867	0
C	1	0.31638	0.61442	0

C	2	0.40696	0.63276	0
C	3	0.42476	0.56216	0
C	4	0.4782	0.59097	0
C	5	0.50731	0.5669	0
C	6	0.48422	0.51309	0
C	7	0.43027	0.4849	0
C	8	0.40138	0.50916	0
C	9	0.36893	0.64967	0
C	10	-0.61442	-0.29804	0
C	11	-0.63276	-0.2258	0
C	12	-0.56216	-0.1374	0
C	13	-0.59097	-0.11277	0
C	14	-0.5669	-0.05959	0
C	15	-0.51309	-0.02887	0
C	16	-0.4849	-0.05463	0
C	17	-0.50916	-0.10778	0
C	18	-0.64967	-0.28074	0
C	19	0.29804	-0.31638	0
C	20	0.2258	-0.40696	0
C	21	0.1374	-0.42476	0
C	22	0.11277	-0.4782	0
C	23	0.05959	-0.50731	0
C	24	0.02887	-0.48422	0
C	25	0.05463	-0.43027	0
C	26	0.10778	-0.40138	0
C	27	0.28074	-0.36893	0
C	28	-0.31638	-0.61442	0
C	29	-0.40696	-0.63276	0
C	30	-0.42476	-0.56216	0
C	31	-0.4782	-0.59097	0
C	32	-0.50731	-0.5669	0
C	33	-0.48422	-0.51309	0
C	34	-0.43027	-0.4849	0
C	35	-0.40138	-0.50916	0
C	36	-0.36893	-0.64967	0
C	37	0.61442	0.29804	0
C	38	0.63276	0.2258	0
C	39	0.56216	0.1374	0
C	40	0.59097	0.11277	0
C	41	0.5669	0.05959	0
C	42	0.51309	0.02887	0
C	43	0.4849	0.05463	0
C	44	0.50916	0.10778	0
C	45	0.64967	0.28074	0
C	46	-0.29804	0.31638	0

C	47	-0.2258	0.40696	0
C	48	-0.1374	0.42476	0
C	49	-0.11277	0.4782	0
C	50	-0.05959	0.50731	0
C	51	-0.02887	0.48422	0
C	52	-0.05463	0.43027	0
C	53	-0.10778	0.40138	0
C	54	-0.28074	0.36893	0
N	1	0.39261	0.58453	0
N	2	-0.58453	-0.19192	0
N	3	0.19192	-0.39261	0
N	4	-0.39261	-0.58453	0
N	5	0.58453	0.19192	0
N	6	-0.19192	0.39261	0
O	1	0.57787	0.90489	1.17665
O	2	-0.90489	-0.32702	1.17665
O	3	0.32702	-0.57787	1.17665
O	4	-0.57787	-0.90489	1.17665
O	5	0.90489	0.32702	1.17665
O	6	-0.32702	0.57787	1.17665
O	7	-0.57787	-0.90489	-1.17665
O	8	0.90489	0.32702	-1.17665
O	9	-0.32702	0.57787	-1.17665
O	10	0.57787	0.90489	-1.17665
O	11	-0.90489	-0.32702	-1.17665
O	12	0.32702	-0.57787	-1.17665
O	13	0.49978	0.7855	1
O	14	-0.7855	-0.28572	1
O	15	0.28572	-0.49978	1
O	16	-0.49978	-0.7855	1
O	17	0.7855	0.28572	1
O	18	-0.28572	0.49978	1
S	1	0.52587	0.85541	1
S	2	-0.85541	-0.32954	1
S	3	0.32954	-0.52587	1
S	4	-0.52587	-0.85541	1
S	5	0.85541	0.32954	1
S	6	-0.32954	0.52587	1

Supplementary Table 4. Eclipsed AA Unit cell parameters and fractional atomic coordinates for NTBAPa-SO_3H calculated based on P6/M group.

Space group			P6/M	
Calculated unit cell			*a* = 23.2201 Å, *b* = 23.2201 Å, *c* = 5.5656 Å,<br>*α* = *β* =90° *γ* = *120*°	
atoms	Atoms number	x	y	z
H	1	2.714358	0.269863	0
H	2	2.784293	0.282669	0
H	3	2.833067	0.42711	0
H	4	2.871803	0.338161	0
H	5	2.958696	0.375932	0
H	6	-0.269863	2.444495	0
H	7	-0.282669	2.501624	0
H	8	-0.42711	2.405957	0
H	9	-0.338161	2.533642	0
H	10	-0.375932	2.582764	0
H	11	-2.444495	-2.714358	0
H	12	-2.501624	-2.784293	0
H	13	-2.405957	-2.833067	0
H	14	-2.533642	-2.871803	0
H	15	-2.582764	-2.958696	0
H	16	0.50131	0.448495	1
H	17	0.453559	0.483359	1
H	18	0.413765	0.520007	1
H	19	0.471172	0.650021	1
H	20	0.3431	0.524246	1
H	21	0.308091	0.563588	1
H	22	0.579323	0.70567	1
H	23	0.433957	0.687149	1
H	24	0.76206	0.411387	1
H	25	-0.448495	0.052816	1
H	26	-0.483359	-0.0298	1
H	27	-0.520007	-0.106242	1
H	28	-0.650021	-0.178849	1
H	29	-0.524246	-0.181146	1
H	30	-0.563588	-0.255496	1
H	31	-0.70567	-0.126348	1
H	32	-0.687149	-0.253192	1
H	33	-0.411387	0.350672	1
H	34	-0.052816	-0.50131	1
H	35	0.0298	-0.453559	1
H	36	0.106242	-0.413765	1
H	37	0.178849	-0.471172	1
H	38	0.181146	-0.3431	1
H	39	0.255496	-0.308091	1
H	40	0.126348	-0.579323	1
H	41	0.253192	-0.433957	1

H	42	-0.350672	-0.76206	1
C	1	2.714425	0.337857	0
C	2	2.731979	0.304155	0
C	3	2.778556	0.31098	0
C	4	2.817564	0.356275	0
C	5	2.806462	0.392288	0
C	6	2.75895	0.381485	0
C	7	2.864346	0.365091	0
C	8	2.946286	0.433306	0
C	9	2.974581	0.412019	0
C	10	3.024419	0.440215	0
C	11	-0.337857	2.376568	0
C	12	-0.304155	2.427824	0
C	13	-0.31098	2.467576	0
C	14	-0.356275	2.461289	0
C	15	-0.392288	2.414174	0
C	16	-0.381485	2.377465	0
C	17	-0.365091	2.499255	0
C	18	-0.433306	2.51298	0
C	19	-0.412019	2.562562	0
C	20	-0.440215	2.584204	0
C	21	-2.376568	-2.714425	0
C	22	-2.427824	-2.731979	0
C	23	-2.467576	-2.778556	0
C	24	-2.461289	-2.817564	0
C	25	-2.414174	-2.806462	0
C	26	-2.377465	-2.75895	0
C	27	-2.499255	-2.864346	0
C	28	-2.51298	-2.946286	0
C	29	-2.562562	-2.974581	0
C	30	-2.584204	-3.024419	0
C	31	0.509606	0.555557	1
C	32	0.489196	0.505704	1
C	33	0.517324	0.484683	1
C	34	0.434622	0.555924	1
C	35	0.411673	0.582482	1
C	36	0.434916	0.631481	1
C	37	0.408866	0.655458	1
C	38	0.358428	0.639108	1
C	39	0.340209	0.588201	1
C	40	0.362693	0.560688	1
C	41	-0.555557	-0.045952	1
C	42	-0.505704	-0.016509	1
C	43	-0.484683	0.032641	1
C	44	-0.555924	-0.121302	1
C	45	-0.582482	-0.170809	1
C	46	-0.631481	-0.196565	1
C	47	-0.655458	-0.246592	1
C	48	-0.639108	-0.28068	1

C	49	-0.588201	-0.247991	1
C	50	-0.560688	-0.197996	1
C	51	0.045952	-0.509606	1
C	52	0.016509	-0.489196	1
C	53	-0.032641	-0.517324	1
C	54	0.121302	-0.434622	1
C	55	0.170809	-0.411673	1
C	56	0.196565	-0.434916	1
C	57	0.246592	-0.408866	1
C	58	0.28068	-0.358428	1
C	59	0.247991	-0.340209	1
C	60	0.197996	-0.362693	1
N	1	2.898948	0.410562	0
N	2	-0.410562	2.488386	0
N	3	-2.488386	-2.898948	0
N	4	3.666667	0.333333	0
N	5	0.482334	0.576063	1
N	6	-0.576063	-0.093729	1
N	7	0.093729	-0.482334	1
N	8	0.333333	0.666667	1
O	1	0.624345	0.664491	1.236545
O	2	-0.664491	-0.040146	1.236545
O	3	0.040146	-0.624345	1.236545
O	4	0.624345	0.664491	-1.236545
O	5	-0.664491	-0.040146	-1.236545
O	6	0.040146	-0.624345	-1.236545
O	7	0.556683	0.671619	1
O	8	-0.671619	-0.114936	1
O	9	0.114936	-0.556683	1
S	1	0.590593	0.646061	1
S	2	-0.646061	-0.055468	1
S	3	0.055468	-0.590593	1

Supplementary Table 5. Eclipsed AA Unit cell parameters and fractional atomic coordinates for TFPBPa-SO_3H calculated based on P6/M group.

Space group			P6/M	
Calculated unit cell			*a* = 23.2201 Å, *b* = 23.2201 Å, *c* = 5.5656 Å, *α* = *β* =90° *γ* = *120°*	
atoms	Atoms number	x	y	z
H	1	0.406832	0.71632	0.5
H	2	0.592004	-0.90147	0.5
H	3	0.683825	-0.760988	0.5
H	4	0.651604	-0.83354	0.5
H	5	0.563407	-0.77141	0.5
H	6	0.533101	-0.843635	0.5
H	7	0.563665	-0.966212	0.5
H	8	0.698992	1.408101	0.5
H	9	0.549819	1.587599	0.5
H	10	0.434423	1.47446	0.5
H	11	0.51624	1.619354	0.5
H	12	0.496786	1.674084	0.5
H	13	0.44936	1.698144	0.5
H	14	0.398269	1.549414	0.5
H	15	0.349398	1.571574	0.5
H	16	0.666862	0.561906	0.5
H	17	-0.71632	-0.309488	0.5
H	18	0.90147	1.493474	0.5
H	19	0.760988	1.444813	0.5
H	20	0.83354	1.485144	0.5
H	21	0.77141	1.334817	0.5
H	22	0.843635	1.376735	0.5
H	23	0.966212	1.529877	0.5
H	24	-1.408101	-0.709109	0.5
H	25	-1.587599	-1.03778	0.5
H	26	-1.47446	-1.040038	0.5
H	27	-1.619354	-1.103113	0.5
H	28	-1.674084	-1.177298	0.5
H	29	-1.698144	-1.248784	0.5
H	30	-1.549414	-1.151145	0.5
H	31	-1.571574	-1.222176	0.5
H	32	-0.561906	0.104956	0.5
H	33	0.309488	-0.406832	0.5
H	34	-1.493474	-0.592004	0.5
H	35	-1.444813	-0.683825	0.5
H	36	-1.485144	-0.651604	0.5
H	37	-1.334817	-0.563407	0.5
H	38	-1.376735	-0.533101	0.5
H	39	-1.529877	-0.563665	0.5
H	40	0.709109	-0.698992	0.5
H	41	1.03778	-0.549819	0.5

H	42	1.040038	-0.434423	0.5
H	43	1.103113	-0.51624	0.5
H	44	1.177298	-0.496786	0.5
H	45	1.248784	-0.44936	0.5
H	46	1.151145	-0.398269	0.5
H	47	1.222176	-0.349398	0.5
H	48	-0.104956	-0.666862	0.5
C	1	0.69075	-0.68502	0.5
C	2	0.647751	-0.710396	0.5
C	3	0.571328	-0.889529	0.5
C	4	0.589822	-0.844434	0.5
C	5	0.627561	-0.757069	0.5
C	6	0.650953	-0.777088	0.5
C	7	0.632389	-0.819751	0.5
C	8	0.584364	-0.782869	0.5
C	9	0.566045	-0.825532	0.5
C	10	0.510219	-0.958155	0.5
C	11	0.530755	-0.980802	0.5
C	12	0.46706	-0.979789	0.5
C	13	0.531939	1.55465	0.5
C	14	0.467584	1.491136	0.5
C	15	0.488847	1.533916	0.5
C	16	0.483048	1.596875	0.5
C	17	0.451389	1.610069	0.5
C	18	0.464395	0.651776	0.5
C	19	0.409382	1.58199	0.5
C	20	0.380908	1.595457	0.5
C	21	0.436161	1.665473	0.5
C	22	0.393317	1.637766	0.5
C	23	0.362349	1.652688	0.5
C	24	0.319778	1.624946	0.5
C	25	0.68502	1.37577	0.5
C	26	0.710396	1.358147	0.5
C	27	0.889529	1.460857	0.5
C	28	0.844434	1.434257	0.5
C	29	0.757069	1.384631	0.5
C	30	0.777088	1.428041	0.5
C	31	0.819751	1.452139	0.5
C	32	0.782869	1.367233	0.5
C	33	0.825532	1.391577	0.5
C	34	0.958155	1.468373	0.5
C	35	0.980802	1.511557	0.5
C	36	0.979789	1.446849	0.5
C	37	-1.55465	-1.022711	0.5
C	38	-1.491136	-1.023552	0.5
C	39	-1.533916	-1.045069	0.5
C	40	-1.596875	-1.113826	0.5
C	41	-1.610069	-1.15868	0.5
C	42	-0.651776	-0.187382	0.5

C	43	-1.58199	-1.172608	0.5
C	44	-1.595457	-1.21455	0.5
C	45	-1.665473	-1.229312	0.5
C	46	-1.637766	-1.244449	0.5
C	47	-1.652688	-1.290339	0.5
C	48	-1.624946	-1.305167	0.5
C	49	-1.37577	-0.69075	0.5
C	50	-1.358147	-0.647751	0.5
C	51	-1.460857	-0.571328	0.5
C	52	-1.434257	-0.589822	0.5
C	53	-1.384631	-0.627561	0.5
C	54	-1.428041	-0.650953	0.5
C	55	-1.452139	-0.632389	0.5
C	56	-1.367233	-0.584364	0.5
C	57	-1.391577	-0.566045	0.5
C	58	-1.468373	-0.510219	0.5
C	59	-1.511557	-0.530755	0.5
C	60	-1.446849	-0.46706	0.5
C	61	1.022711	-0.531939	0.5
C	62	1.023552	-0.467584	0.5
C	63	1.045069	-0.488847	0.5
C	64	1.113826	-0.483048	0.5
C	65	1.15868	-0.451389	0.5
C	66	0.187382	-0.464395	0.5
C	67	1.172608	-0.409382	0.5
C	68	1.21455	-0.380908	0.5
C	69	1.229312	-0.436161	0.5
C	70	1.244449	-0.393317	0.5
C	71	1.290339	-0.362349	0.5
C	72	1.305167	-0.319778	0.5
N	1	0.531804	-0.91391	0.5
N	2	0.467707	1.552714	0.5
N	3	0.91391	1.445715	0.5
N	4	-1.552714	-1.085007	0.5
N	5	-1.445715	-0.531804	0.5
N	6	1.085007	-0.467707	0.5
O	1	0.624466	0.592661	0.721778
O	2	-0.592661	0.031805	0.721778
O	3	-0.031805	-0.624466	0.721778
O	4	0.624466	0.592661	-0.721778
O	5	-0.592661	0.031805	-0.721778
O	6	-0.031805	-0.624466	-0.721778
O	7	0.638171	0.539113	0.5
O	8	-0.539113	0.099058	0.5
O	9	-0.099058	-0.638171	0.5
S	1	0.608553	0.562266	0.5
S	2	-0.562266	0.046287	0.5
S	3	-0.046287	-0.608553	0.5

Supplementary Table 6. Eclipsed AA Unit cell parameters and fractional atomic coordinates for CABPTZPa-SO_3H calculated based on P6/M group.

Space group			P6/M	
Calculated unit cell			*a* = 23.2201 Å, *b* = 23.2201 Å, *c* = 5.5656 Å, *α* = *β* =90° *γ* = *120°*	
atoms	Atoms number	x	y	z
H	1	1.409865	-0.269367	0
H	2	1.439197	-0.217075	0
H	3	1.359358	-0.211201	0
H	4	1.3308	-0.263547	0
H	5	1.523249	-0.024121	0
H	6	1.547973	0.028977	0
H	7	1.464388	-0.171563	0
H	8	1.49293	-0.119048	0
H	9	1.413966	-0.113425	0
H	10	1.384527	-0.165693	0
H	11	2.581995	0.509239	0
H	12	0.269367	1.679232	0
H	13	0.217075	1.656273	0
H	14	0.211201	1.570559	0
H	15	0.263547	1.594348	0
H	16	0.024121	1.54737	0
H	17	-0.028977	1.518996	0
H	18	0.171563	1.635951	0
H	19	0.119048	1.611978	0
H	20	0.113425	1.527391	0
H	21	0.165693	1.55022	0
H	22	-0.509239	2.072756	0
H	23	-1.679232	-1.409865	0
H	24	-1.656273	-1.439197	0
H	25	-1.570559	-1.359358	0
H	26	-1.594348	-1.3308	0
H	27	-1.54737	-1.523249	0
H	28	-1.518996	-1.547973	0
H	29	-1.635951	-1.464388	0
H	30	-1.611978	-1.49293	0
H	31	-1.527391	-1.413966	0
H	32	-1.55022	-1.384527	0
H	33	-2.072756	-2.581995	0
H	34	0.431501	0.502121	1
H	35	0.561363	0.535697	1
H	36	0.624135	0.501383	1
H	37	0.645454	0.470392	1
H	38	0.557452	0.393602	1
H	39	0.537183	0.425411	1
H	40	0.663453	0.443114	1
H	41	0.683742	0.411402	1

H	42	0.596629	0.335387	1
H	43	0.575486	0.366355	1
H	44	0.540968	0.618751	1
H	45	-0.502121	-0.07062	1
H	46	-0.535697	0.025665	1
H	47	-0.501383	0.122752	1
H	48	-0.470392	0.175062	1
H	49	-0.393602	0.163851	1
H	50	-0.425411	0.111771	1
H	51	-0.443114	0.220339	1
H	52	-0.411402	0.27234	1
H	53	-0.335387	0.261242	1
H	54	-0.366355	0.209131	1
H	55	-0.618751	-0.077784	1
H	56	0.07062	-0.431501	1
H	57	-0.025665	-0.561363	1
H	58	-0.122752	-0.624135	1
H	59	-0.175062	-0.645454	1
H	60	-0.163851	-0.557452	1
H	61	-0.111771	-0.537183	1
H	62	-0.220339	-0.663453	1
H	63	-0.27234	-0.683742	1
H	64	-0.261242	-0.596629	1
H	65	-0.209131	-0.575486	1
H	66	0.077784	-0.540968	1
C	1	1.367936	-0.270788	0
C	2	1.398727	-0.256688	0
C	3	1.415751	-0.226002	0
C	4	1.402637	-0.208066	0
C	5	1.371479	-0.222744	0
C	6	1.354547	-0.253436	0
C	7	1.478635	-0.030819	0
C	8	1.509676	-0.014062	0
C	9	1.524142	0.016633	0
C	10	2.603861	0.428351	0
C	11	1.452252	-0.160035	0
C	12	1.469156	-0.129338	0
C	13	1.455757	-0.112056	0
C	14	1.425015	-0.126088	0
C	15	1.407972	-0.156776	0
C	16	2.566806	0.485819	0
C	17	1.654802	0.351282	0
C	18	0.270788	1.638725	0
C	19	0.256688	1.655415	0
C	20	0.226002	1.641754	0
C	21	0.208066	1.610703	0
C	22	0.222744	1.594223	0
C	23	0.253436	1.607983	0
C	24	0.030819	1.509454	0

C	25	0.014062	1.523737	0
C	26	-0.016633	1.507509	0
C	27	-0.428351	2.17551	0
C	28	0.160035	1.612287	0
C	29	0.129338	1.598494	0
C	30	0.112056	1.567813	0
C	31	0.126088	1.551103	0
C	32	0.156776	1.564748	0
C	33	-0.485819	2.080987	0
C	34	-0.351282	1.30352	0
C	35	-1.638725	-1.367936	0
C	36	-1.655415	-1.398727	0
C	37	-1.641754	-1.415751	0
C	38	-1.610703	-1.402637	0
C	39	-1.594223	-1.371479	0
C	40	-1.607983	-1.354547	0
C	41	-1.509454	-1.478635	0
C	42	-1.523737	-1.509676	0
C	43	-1.507509	-1.524142	0
C	44	-2.17551	-2.603861	0
C	45	-1.612287	-1.452252	0
C	46	-1.598494	-1.469156	0
C	47	-1.567813	-1.455757	0
C	48	-1.551103	-1.425015	0
C	49	-1.564748	-1.407972	0
C	50	-2.080987	-2.566806	0
C	51	-1.30352	-1.654802	0
C	52	0.346719	0.65009	1
C	53	0.404193	0.578914	1
C	54	0.446013	0.525645	1
C	55	0.521586	0.537379	1
C	56	0.537663	0.522617	1
C	57	0.523205	0.49168	1
C	58	0.579099	0.465903	1
C	59	0.609591	0.477747	1
C	60	0.621808	0.459417	1
C	61	0.572998	0.416829	1
C	62	0.560904	0.435268	1
C	63	0.617064	0.408374	1
C	64	0.647911	0.419887	1
C	65	0.660012	0.401459	1
C	66	0.6419	0.3708	1
C	67	0.611334	0.358982	1
C	68	0.599132	0.377323	1
C	69	-0.65009	-0.303371	1
C	70	-0.578914	-0.174721	1
C	71	-0.525645	-0.079632	1
C	72	-0.537379	-0.015793	1
C	73	-0.522617	0.015046	1

C	74	-0.49168	0.031525	1
C	75	-0.465903	0.113196	1
C	76	-0.477747	0.131844	1
C	77	-0.459417	0.16239	1
C	78	-0.416829	0.156169	1
C	79	-0.435268	0.125636	1
C	80	-0.408374	0.20869	1
C	81	-0.419887	0.228024	1
C	82	-0.401459	0.258553	1
C	83	-0.3708	0.2711	1
C	84	-0.358982	0.252352	1
C	85	-0.377323	0.221809	1
C	86	0.303371	-0.346719	1
C	87	0.174721	-0.404193	1
C	88	0.079632	-0.446013	1
C	89	0.015793	-0.521586	1
C	90	-0.015046	-0.537663	1
C	91	-0.031525	-0.523205	1
C	92	-0.113196	-0.579099	1
C	93	-0.131844	-0.609591	1
C	94	-0.16239	-0.621808	1
C	95	-0.156169	-0.572998	1
C	96	-0.125636	-0.560904	1
C	97	-0.20869	-0.617064	1
C	98	-0.228024	-0.647911	1
C	99	-0.258553	-0.660012	1
C	100	-0.2711	-0.6419	1
C	101	-0.252352	-0.611334	1
C	102	-0.221809	-0.599132	1
N	1	1.462896	-0.062603	0
N	2	1.637094	0.321564	0
N	3	0.062603	1.525499	0
N	4	-0.321564	1.31553	0
N	5	-1.525499	-1.462896	0
N	6	-1.31553	-1.637094	0
N	7	0.363054	0.679945	1
N	8	0.538506	0.475316	1
N	9	-0.679945	-0.31689	1
N	10	-0.475316	0.06319	1
N	11	0.31689	-0.363054	1
N	12	-0.06319	-0.538506	1
O	1	0.56392	0.589015	1.220379
O	2	-0.589015	-0.025095	1.220379
O	3	0.025095	-0.56392	1.220379
O	4	0.56392	0.589015	-1.220379
O	5	-0.589015	-0.025095	-1.220379
O	6	0.025095	-0.56392	-1.220379
O	7	0.524826	0.597995	1
O	8	-0.597995	-0.073169	1

O	9	0.073169	-0.524826	1
S	1	0.542072	0.577245	1
S	2	-0.577245	-0.035172	1
S	3	0.035172	-0.542072	1

Supplementary Table 7. Synthesis condition of iCOF nanosheets

Sample	Heterogeneous assembly		Reaction temperature / °C	Time/ days
TpPa-SO_3H	Pa-SO_3H/H_2O	Tp/ n-caprylic acid	15	3
TpPa-SO_3H(2)	Pa-SO_3H(2)/H_2O	Tp/ n-caprylic acid	60	20
TpBd-SO_3H	Bd-SO_3H/H_2O	Tp/ n-caprylic acid	60	20
	Homogeneous assembly			
NTBAPa-SO_3H	Pa-SO_3H/DMSO	NTBA/ DMSO	120	7
TFPBPa-SO_3H	Pa-SO_3H/DMSO	TFPB/ DMSO	120	7
CABPTZPa-SO_3H	Pa-SO_3H/DMSO	CABPTZP/ DMSO	120	7

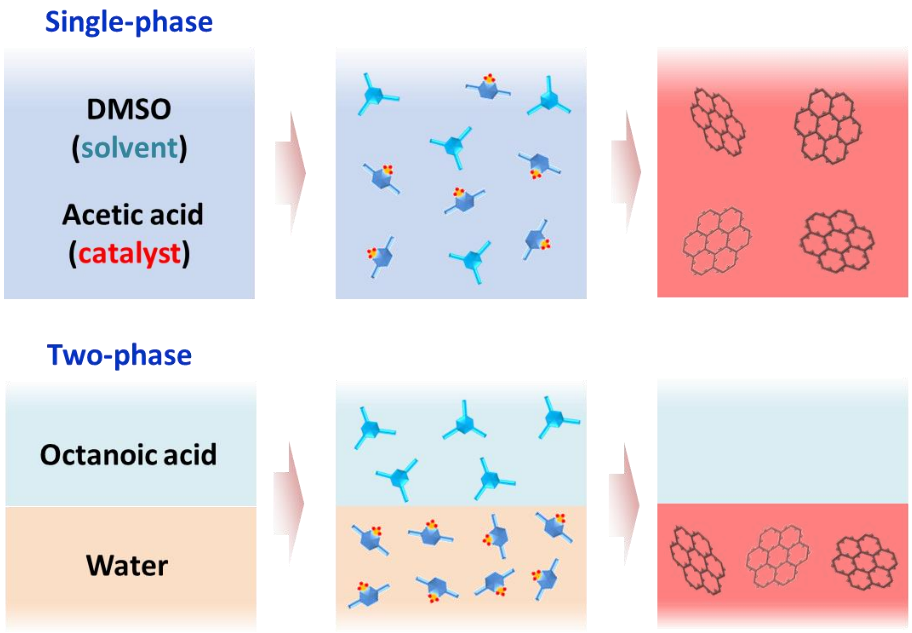

Supplementary Figure 3. Synthesis of iCOF nanosheets by single-phase and two-phase system.

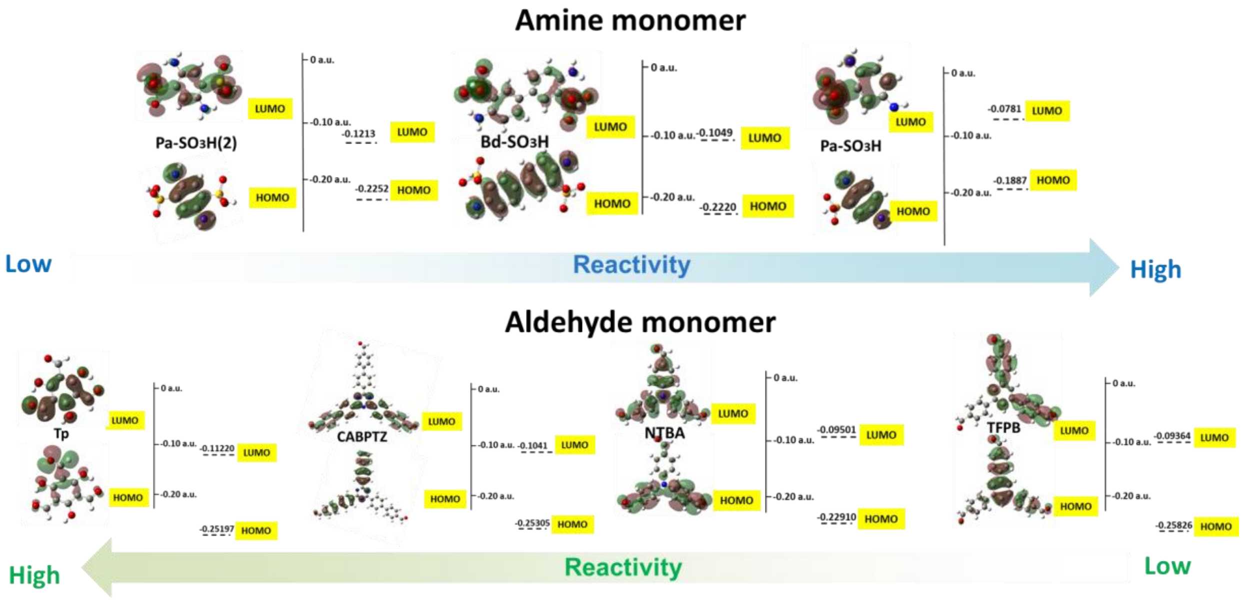

Supplementary Figure 4. The HOMO and LUMO values of the amine and aldehyde monomer.

Note: The HOMO and LUMO values of the amine and aldehyde monomer were calculated in Gaussian 09W package at DFT/B3LYP/6-311G++(d,p) level. During the Schiff base reaction, -NH_2 is the electron donor and –CH=O is the electron acceptor. For ionic amine monomers, the reactivity is promoted with the decrease of HOMO value. For aldehyde monomer, the reactivity is promoted with the increase of LUMO value.

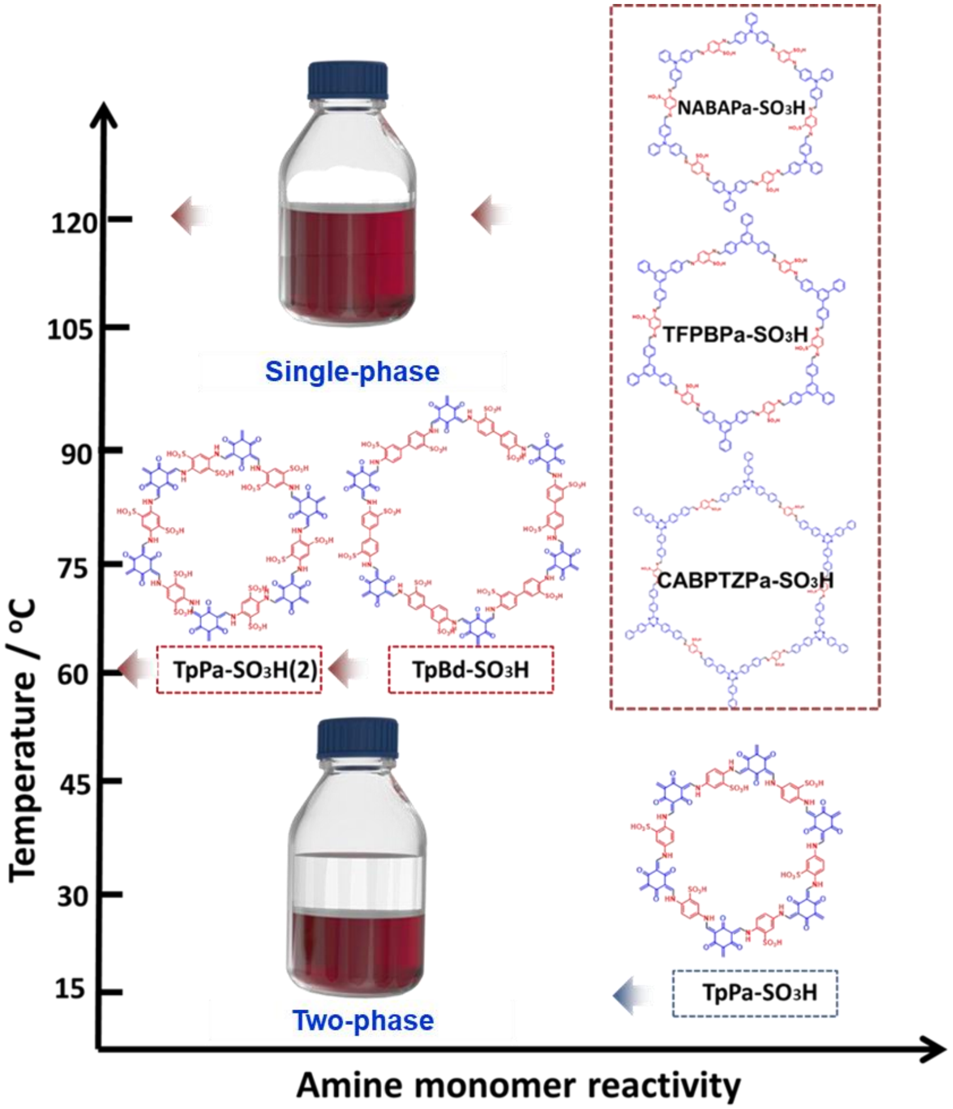

Supplementary Figure 5. Synthesis condition of the iCOF nanosheets.

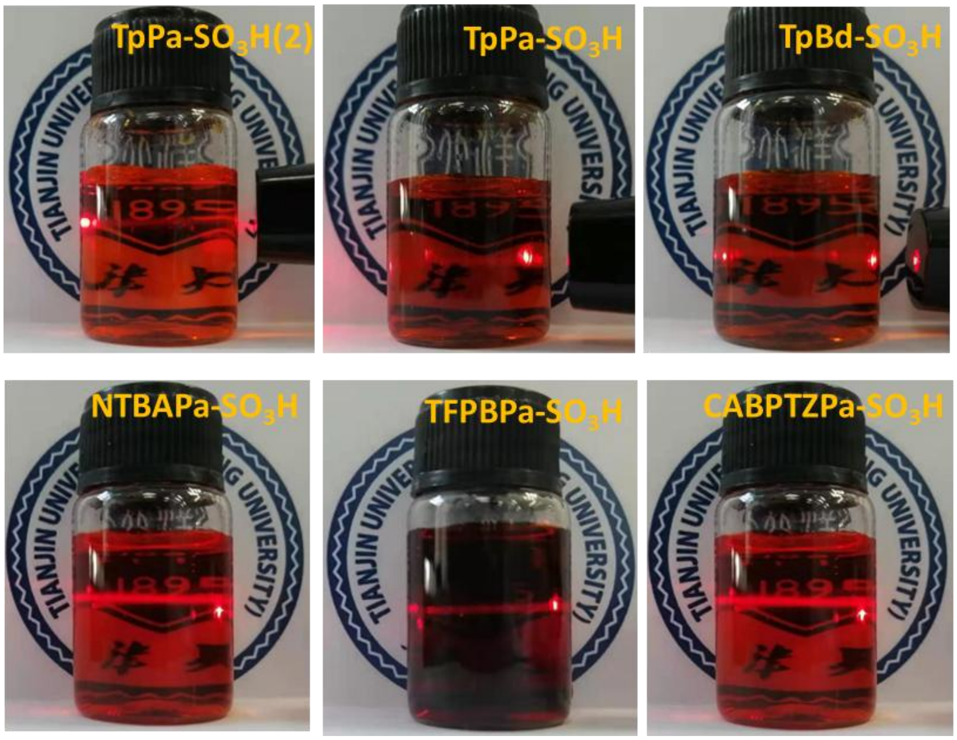

Supplementary Figure 6. The photograph of the iCOF nanosheets suspensions.

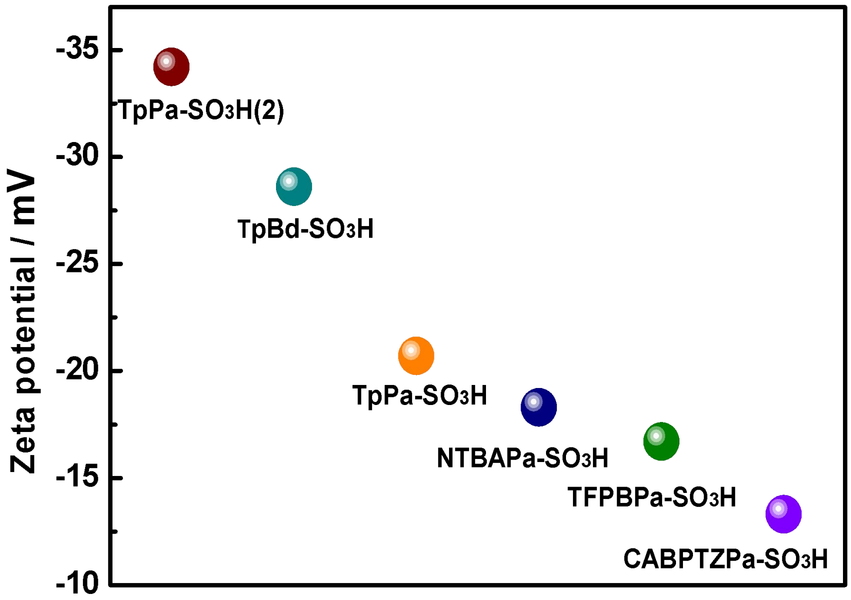

Supplementary Figure 7. The zeta potential of the iCOF nanosheets suspensions.

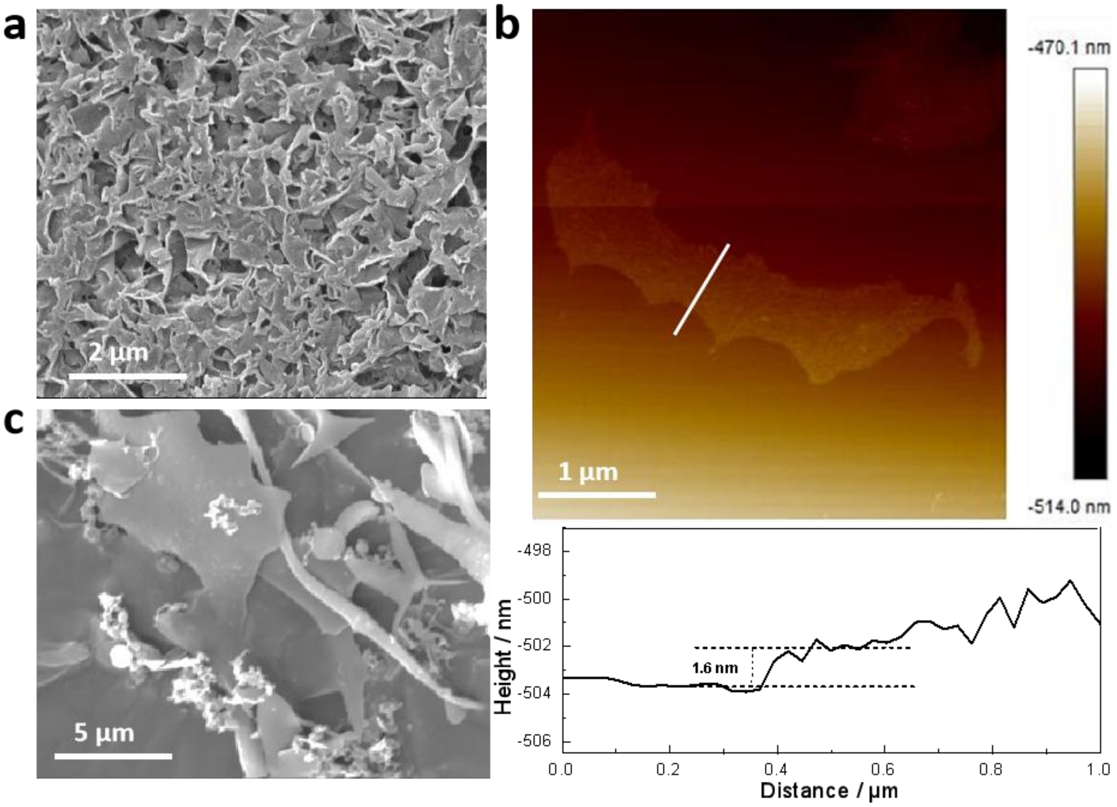

Supplementary Figure 8. SEM images of iCOF nanosheet (a and b). AFM image of iCOF nanosheet (c).

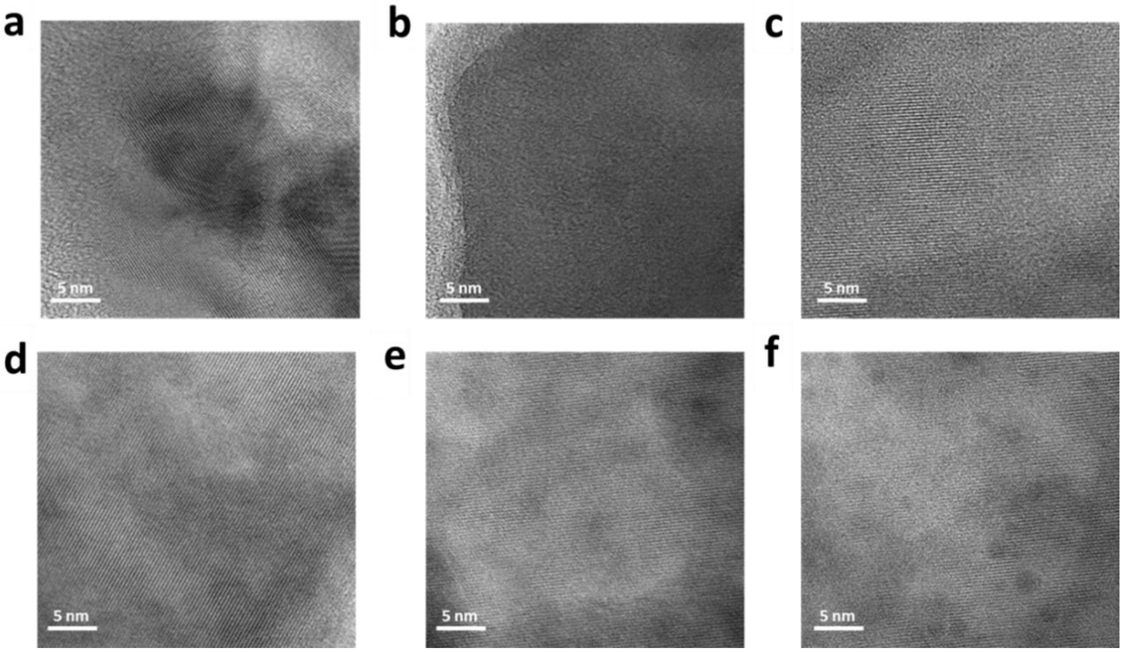

Supplementary Figure 9. HRTEM of the iCOF nanosheets ((a)TpPa-SO_3H(2), (b)TpBd-SO_3H, (c)TpPa-SO_3H, (d)NTBAPa-SO_3H, (e) TFPBPa-SO_3H, (f) CAPBTZPa-SO_3H.

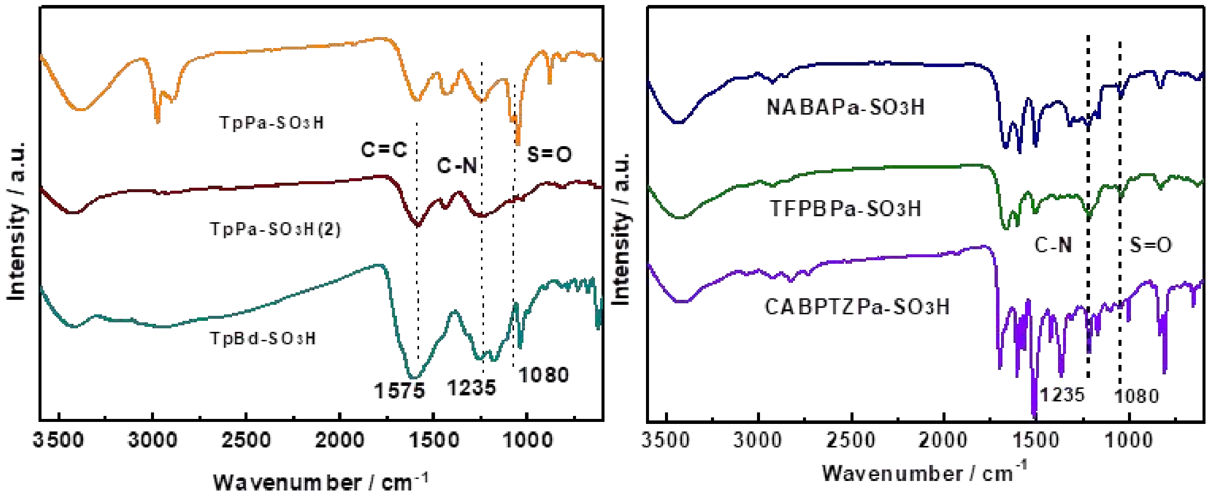

Supplementary Figure 10. The FTIR of the TpPa-SO_3H(2), TpBd-SO_3H,TpPa-SO_3H NABAPa-SO_3H, TFPBPa-SO_3H, CABPTZPa-SO_3H.

Note: The chemical structure of iCOF nanosheets were confirmed by FTIR, as shown in Fig.10. The typical peaks at 1575 and 1235 cm^{-1} can be attributed to C=C and C-N stretching, respectively. The absorption peak at 1080 cm^{-1} is attributed to the symmetric O=S=O stretching vibration of -SO_3H groups in iCOF.

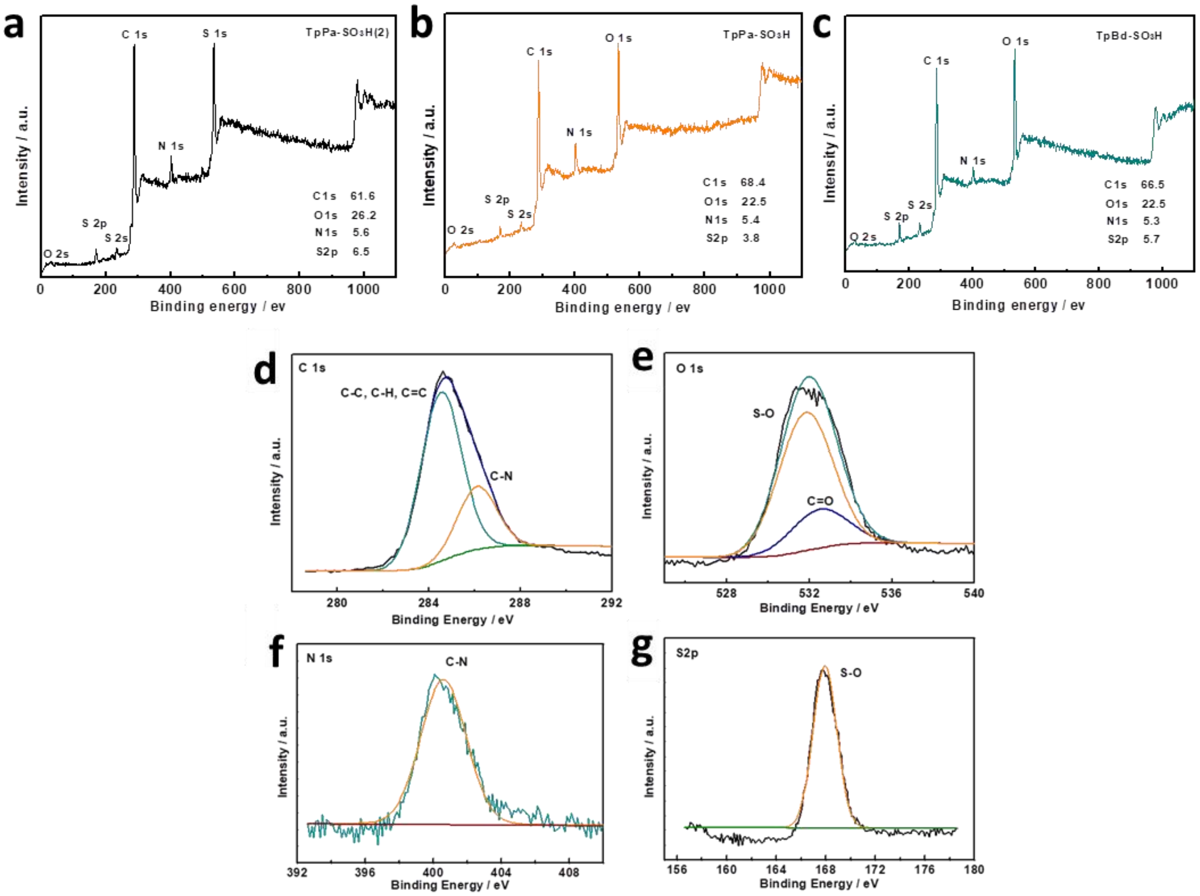

Supplementary Figure 11. The XPS spectra (a, b, and c) and high resolution XPS spectra (d, e, f, and g) of TpPa-SO_3H(2), TpBd-SO_3H and TpPa-SO_3H.

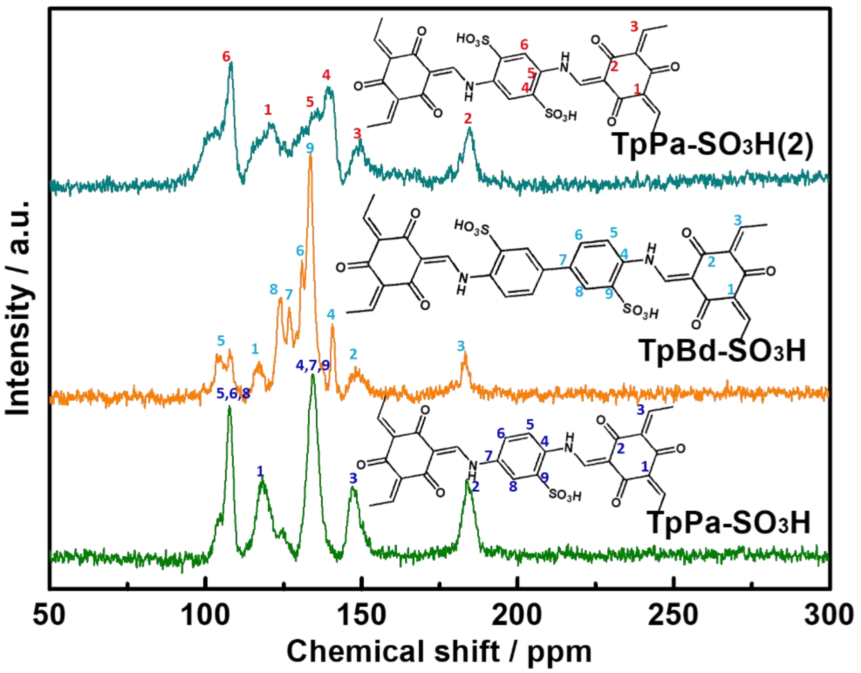

Supplementary Figure 12. The solid-state C-NMR of TpPa-SO_3H(2), TpBd-SO_3H and TpPa-SO_3H.

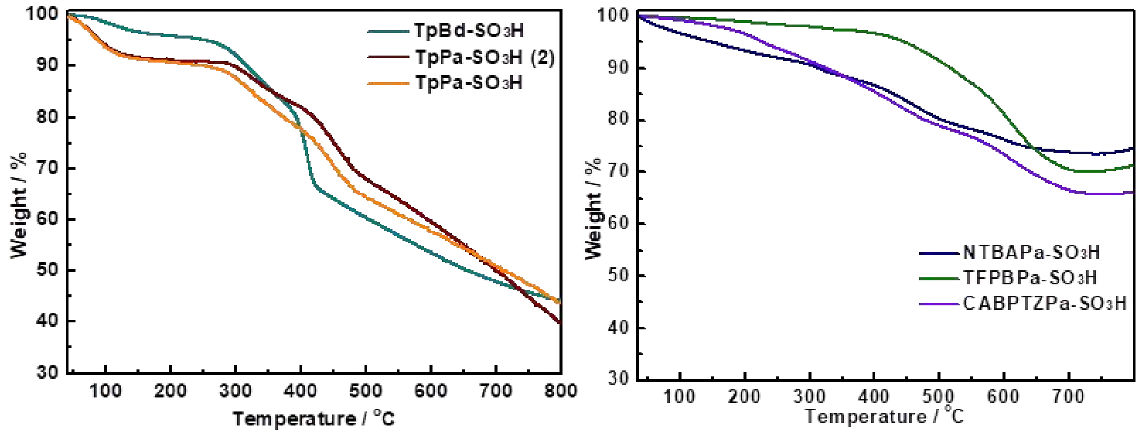

Supplementary Figure 13. The TGA curves of TpPa-SO_3H(2), TpBd-SO_3H and TpPa-SO_3H.

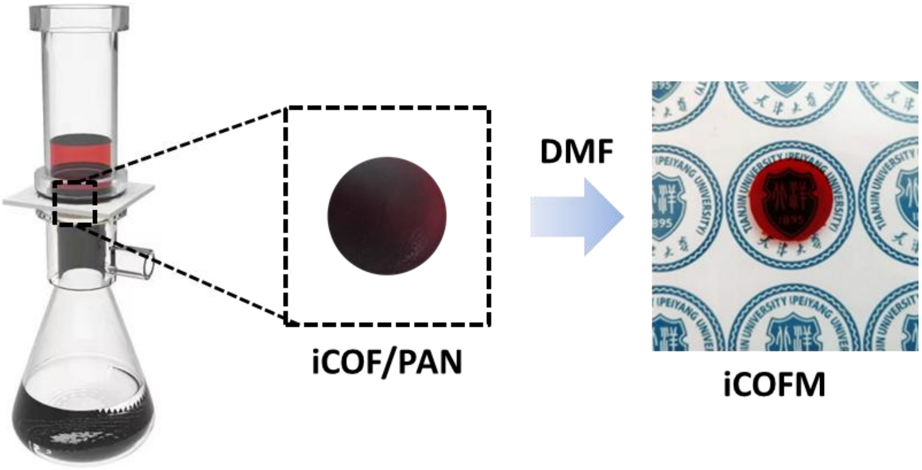

Supplementary Figure 14. The preparation of iCOFMs by vacuum assisted self-assembly method.

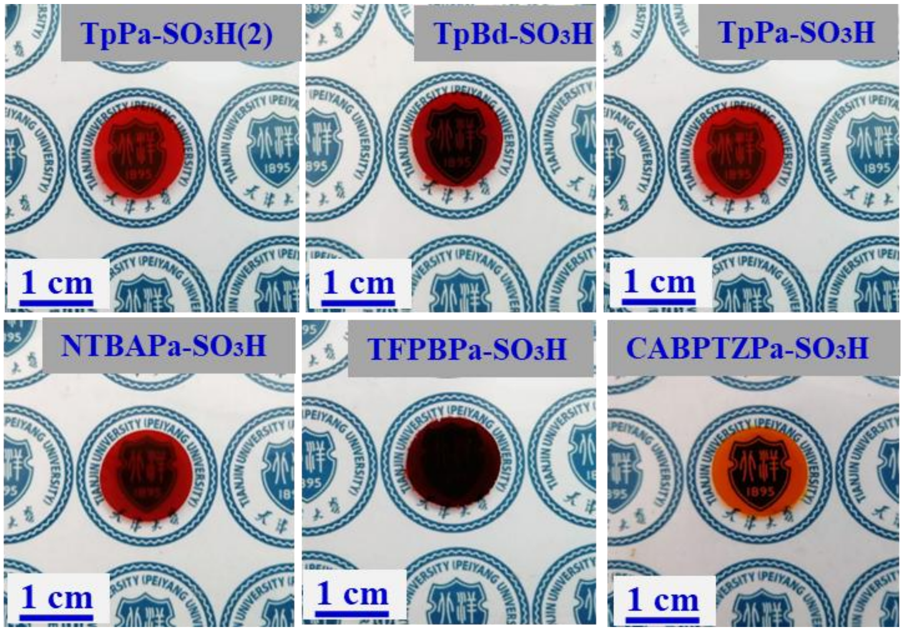

Supplementary Figure 15. The photograph of iCOFMs (TpPa-SO_3H(2), TpBd-SO_3H, TpPa-SO_3H, NTBAPa-SO_3H, TFPBPa-SO_3H, CABPTZPa- SO_3H).

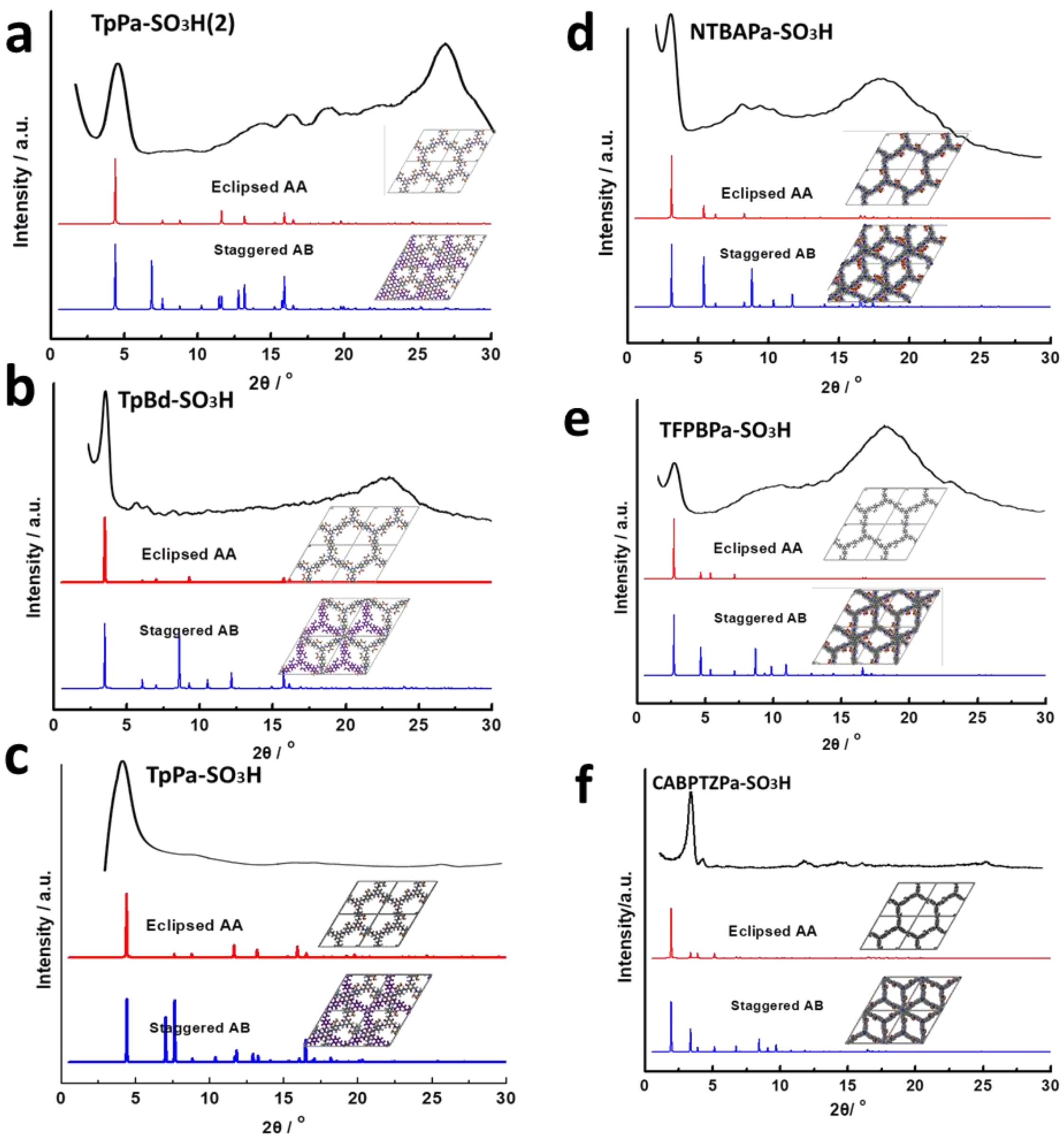

Supplementary Figure 16. The XRD patterns of the TpPa-SO_3H(2) (a), TpBd-SO_3H(b), TpPa-SO_3H (c), NTBAPa-SO_3H (d), TFPBPa-SO_3H (e), CABPTZPa-SO_3H (f).

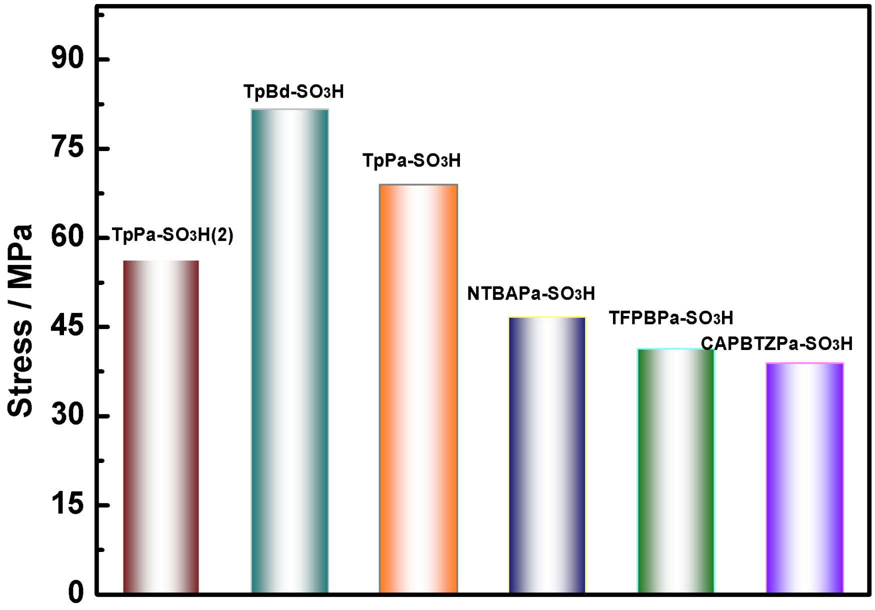

Supplementary Figure 17. The tensile strength of the iCOFM.

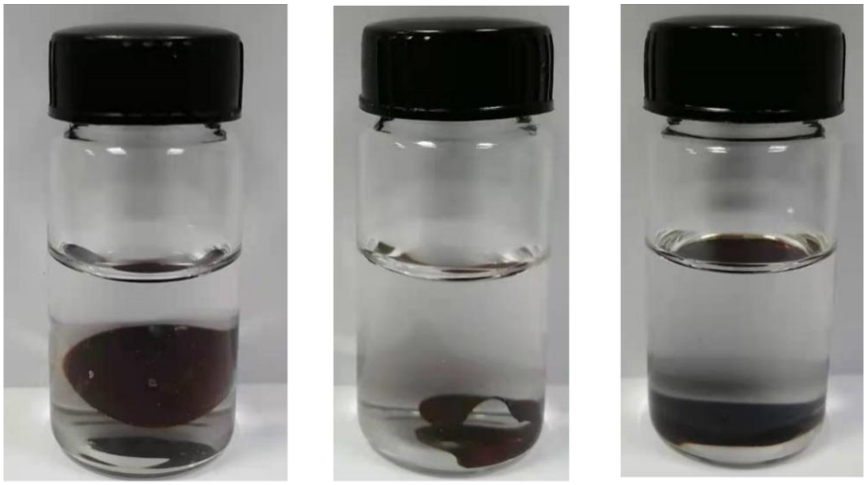

Supplementary Figure 18. The photograph of iCOFMs in water, DMF, 1M H_2SO_4 solution.

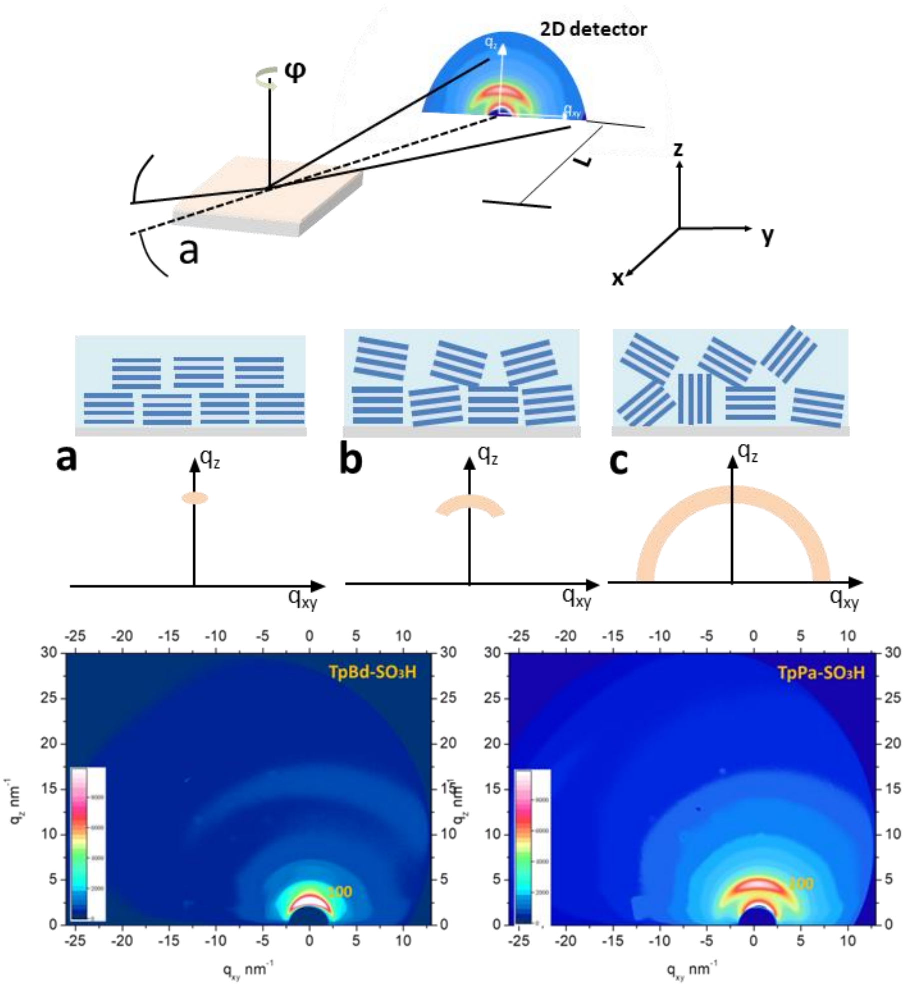

Supplementary Figure 19. GIWAXs data of the TpBd-SO3H and the TpPa-SO3H membrane.

Note: Grazing incidence small-angle X-ray scattering (GISAXS) was used to probe the orientation of the iCOF nanosheets in the membrane. Three kinds of models of the arrangements of the crystallites were shown in Fig.19. Fig.19a represents the randomly oriented arrangements of the crystallites, with no preference for a specific crystallographic orientation with respect to the substrate normal produce rings in the diffraction patterns. Fig.19b represents oriented films with a distribution of crystallite orientations produce arcs of diffracted intensity. Fig.19c represents highly oriented films produce spots or ellipses. The orientation of the assembled iCOF nanosheets can refer to the models. Both iCOFMs displayed arcs of diffracted intensity, indicating that the iCOF nanosheets were parallelly aligned.

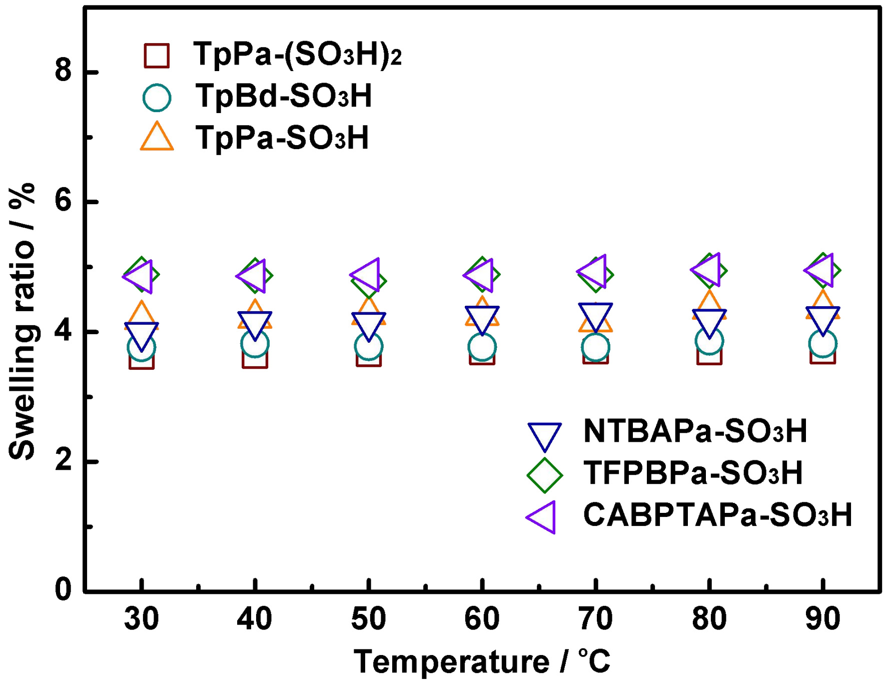

Supplementary Figure 20. Temperature dependent swelling ratio of the membranes.

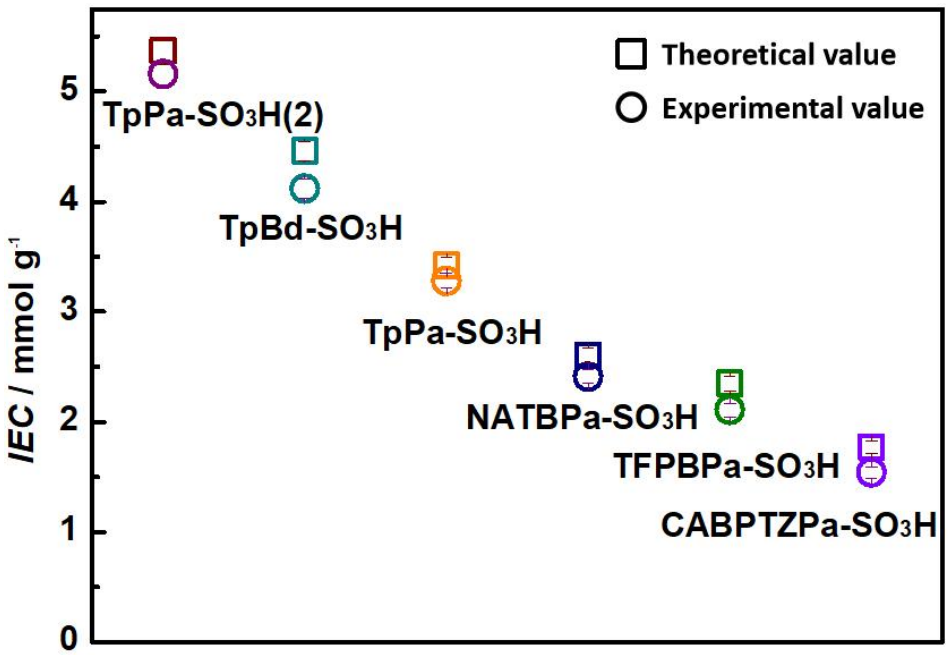

Supplementary Figure 21. The *IEC* values of iCOFMs (the square marks represent the theoretical values and the circle marks represent the experimental values).

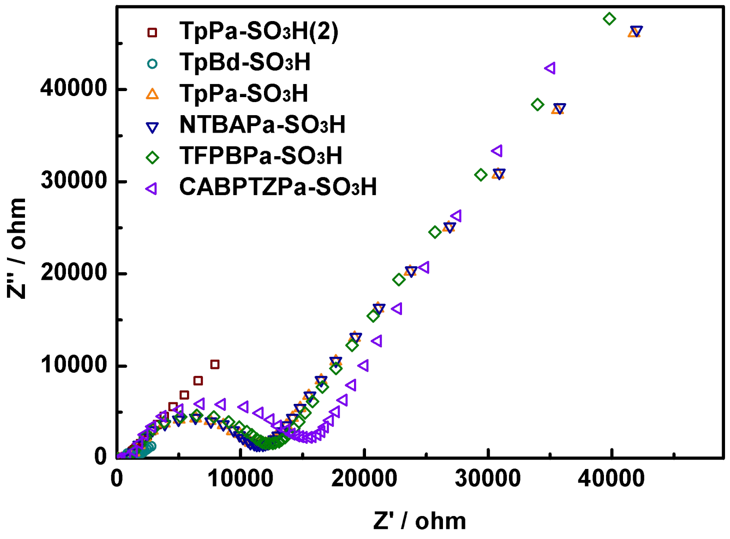

Supplementary Figure 22. Nyquist plots of the COFs at 90 °C under 100% RH.

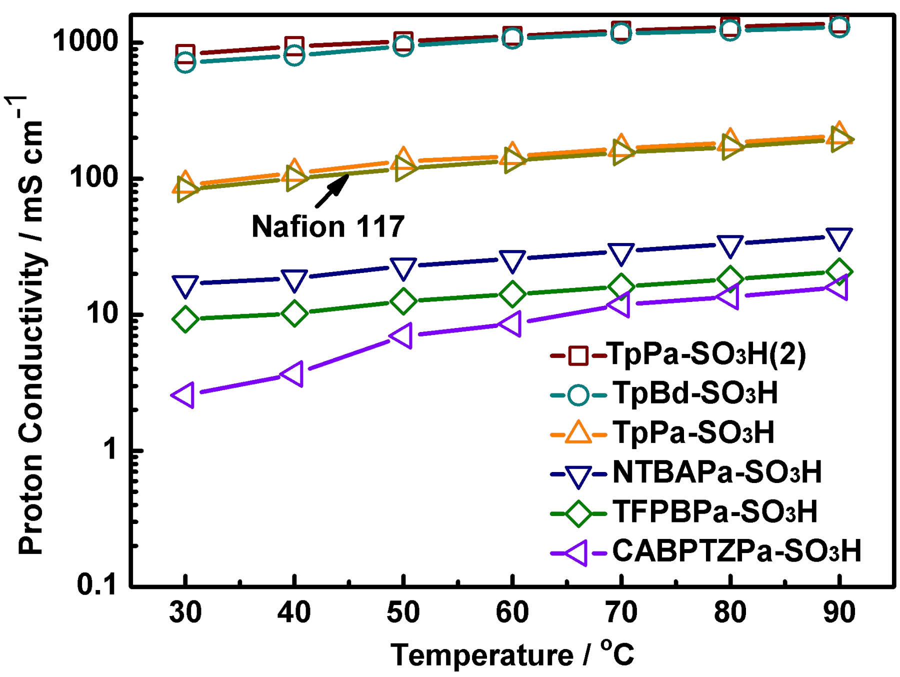

Supplementary Figure 23. Temperature dependent proton conductivities of the membranes under 100% RH.

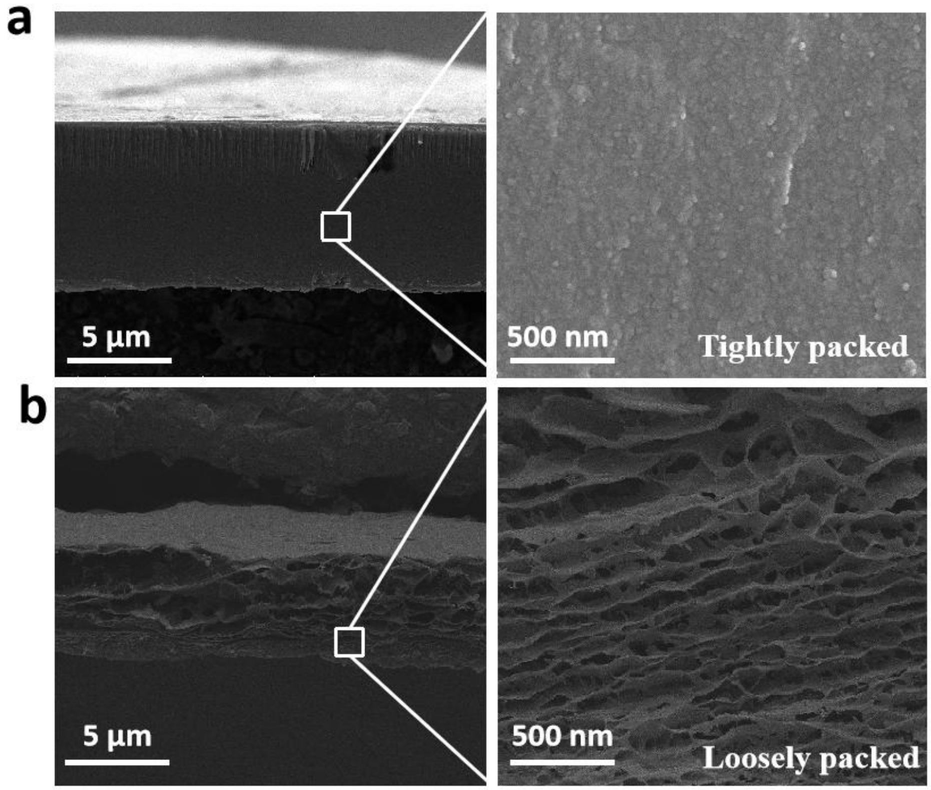

Supplementary Figure 24. SEM images of the cross section of the TpBd-SO_3H prepared by vacuum pressure of -0.08 MPa (a) and -0.025 MPa (b), respectively.

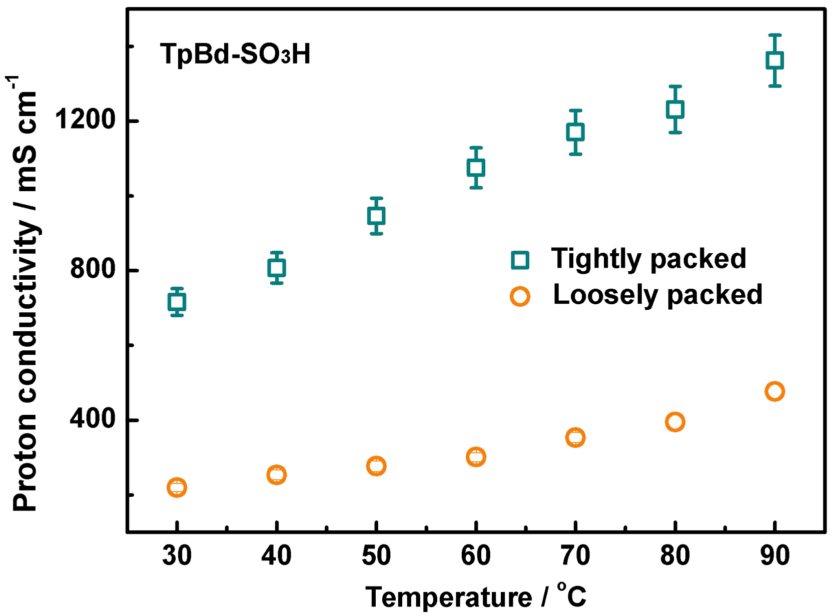

Supplementary Figure 25. Temperature dependent proton conductivities of the TpBd-SO_3H with tightly packed and loosely packed nanosheets.All the error bars in this figure represent the standard deviation of the experiments.

Note: The TpBd-SO_3H with tightly packed and loosely packed nanosheets was prepared by adjusting vacuum pressure. When using high pressure, the TpBd-SO_3H nanosheets are tightly packed. When using low pressure, the TpBd-SO_3H nanosheets are loosely packed.

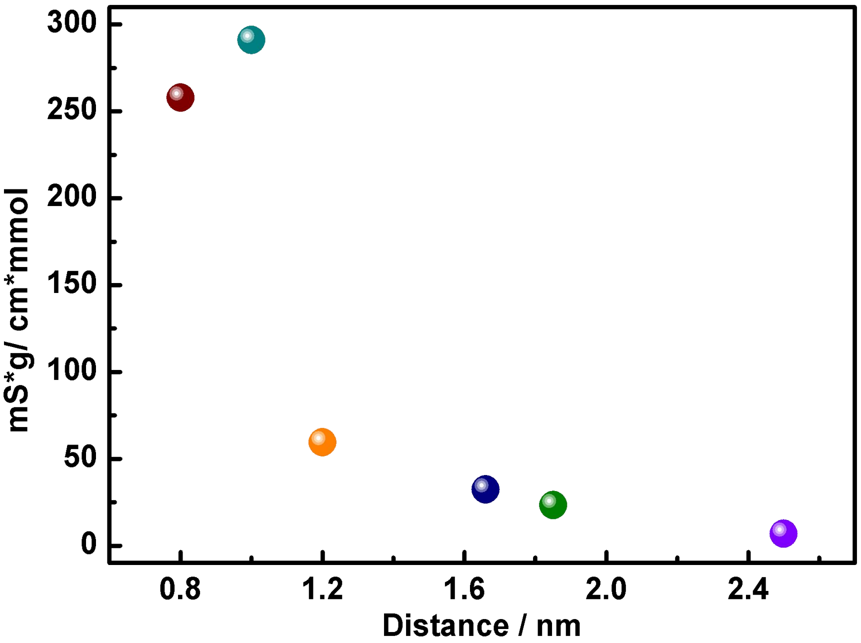

Supplementary Figure 26. -SO_3H group distance dependent normalized proton conductivity of the iCOFMs.

Supplementary Table 8. Proton conductivity, IEC values, and mechanical stability of iCOFMs and as reported in literature

Membrane	IEC values (mmol g^{-1})	Stress (MPa)	Proton conductivity(mS cm^{-1} (Temperature/ ^{o}C)	Reference
PTFE48/SPEEK65	1.44	27.2	183 (90)	1
Aq-PSUT（30%）	1.02	20	180(80)	2
1 wt.% Co-tri MOF/Aquivion®	1.2		149(80)	3
B3	1.68	157	14.98(80)	4
GPS-60	2.06	157	336(80)	5
Su-CNTs/Nafion	0.98	20.6	216 (99)	6
SPPESK-SF-7.5	1.62	31.4	226.7(90)	7
Nafion/ZSNFs		28.18	265 (80)	8
AMA-4.0@Nafion		5.6	158(80)	9
Nafion/CeO2-TiC(0.5 wt%)	0.87	46.9	90(90)	10
PTPBSH-90	2.44	51.2	119(90)	11
Nafion-ZrGdNR-1.5	0.909	22.5	164(80)	12
M-BC	1.75	25	90.2 (80)	13
Nafion-SSA 1.0 wt%	1.31		230.1 (80)	14
GO-g-SPEEK/Nafion-33	1.45	8.6	219(90)	15
PTSA@TpAzo		15	78(80)	16
SGO-Nafion-40		27	110(80)	17
N_U200-2		12.4	171(80)	18
Me-m-SPEEKK	1.78	55.4	173(100)	19
sPBT-6F90	2.19	75.5	150(90)	20
SPP-QP	2.6	34	220 (80)	21
SPEEK/S-UiO-66@GO-10	1.7	53.5	268(70)	22
SPAFT5	1.77	44.3	105(80)	23
SF-Nafion-1	0.932	13	130(80)	24
MSAIT2		41.8	120(80)	25
MIV	0.56	6.25	32(80)	26
SPAES-8-33	1.66	20.51	331(80)	27
S40-N3	1.01		142(100)	28
6F-PAEK-SP22	1.77	29.5	148(100)	29
SPI-5-POSS	1.69	40.8	132(80)	30
C-SPAES-40	2.12	71.5	150.4(80)	31
PEEK-PEMs	3.08	14	431(80)	32
SPAES/ABPBI-GO 1.0	1.57	59.5	152.5(80)	33
SPEKEBI-4	2.14	11	233(80)	34
SPAEK-100	1.49	45.34	159(100)	35
SPAES50	1.84	54.86	211.6 (80)	36
SPEEK/PANTs-3#-10	1.869	57.25	330(70)	37
poly(SHS-ddm)	3.13	32.49	154 (80)	38
SPAES 50	2.01	9.2	181(80)	39

SQNPAEK-2.5	2.69	40.18	317(80)	40
PAQSH-60	1.47	44	50(80)	41
SPES-3	1.51	27	131.4(100)	42
sPBT-PE57.5	2.55	49.2	130(80)	43
SPP-co-PAEK(5/1)	2.38	46	233(60)	44
PEM-OH	1.6	48	100(80)	45
Am-SPEEK-T	1.53	53.8	140 (80)	46
2-SPAES-80	1.73	53	258(80)	47
SPAEK	1.73	49	130(120)	48
IPC-COF	3.2	91.2	380(80)	49
MOG	0.2	--	7.8(80)	50
GO	1.34	39.5	118.3(80)	51
GO/SL-100%	1.81	94.8	346(80)	51
TpPa-SO_3H(2)	5.37	65	1389(90)	This work
TpBd-SO_3H	4.46	70	1302(90)	This work

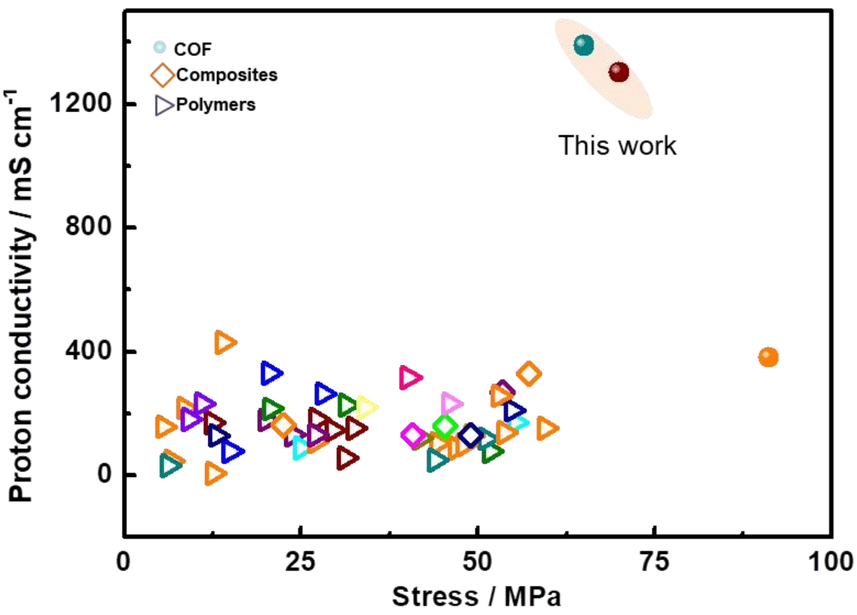

Supplementary Figure 27. The comparison of the mechanical stability and proton conductivity between the iCOFMs and the membranes reported in literatures.

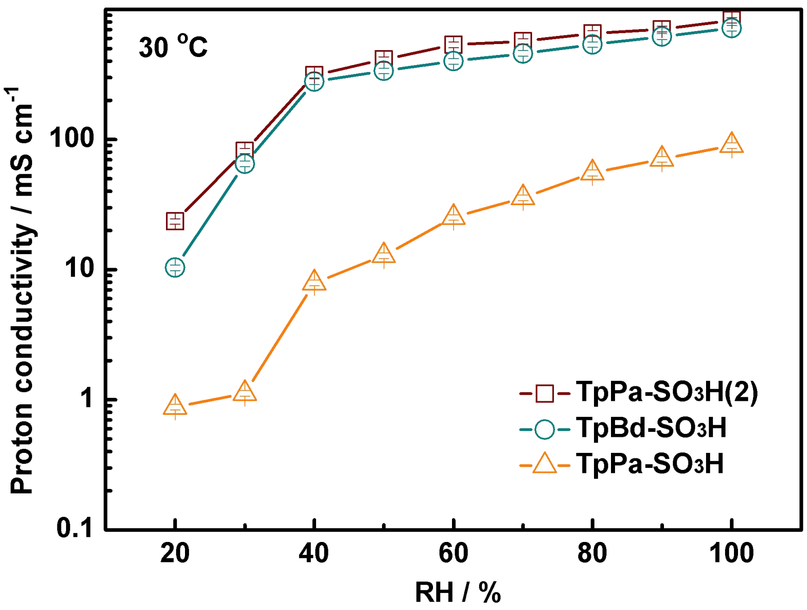

Supplementary Figure 28. Relative humidity dependent in-plane proton conductivities of the iCOFMs at 90 °C.All the error bars in this figure represent the standard deviation of the experiments.

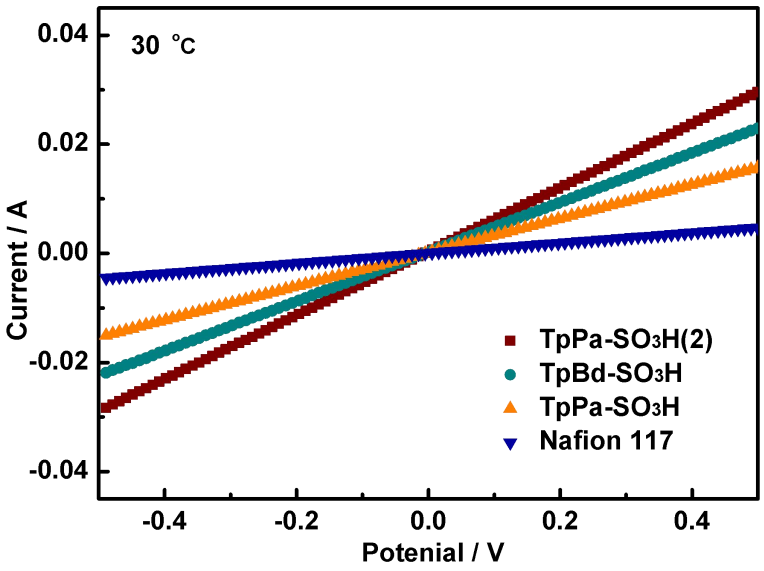

Supplementary Figure 29. I-V curves of the iCOFMs and Nafion 117.

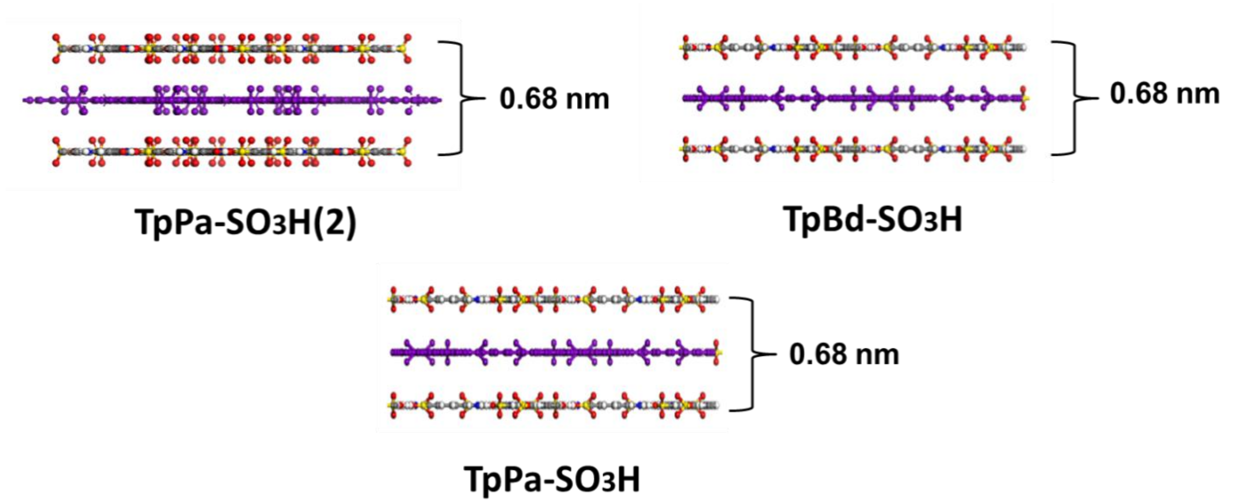

Supplementary Figure 30. The $–SO_3H$ group distance between the eclipsed AA stacked iCOF nanosheets.

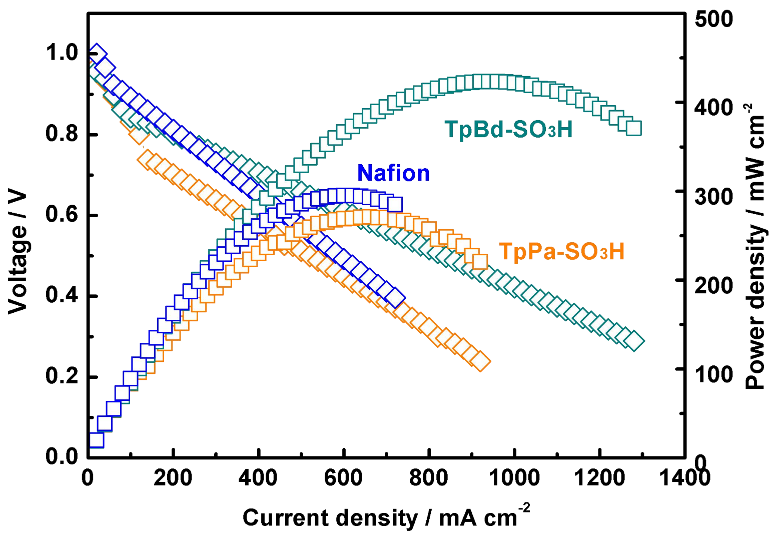

Supplementary Figure 31. Single fuel cell performance of the TpBd-SO_3H, TpPa-SO_3H, and Nafion under the same condition.

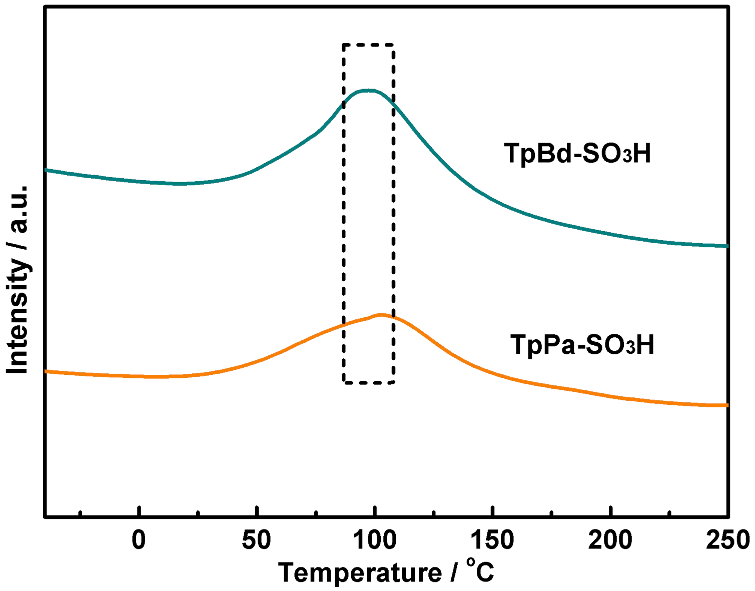

Supplementary Figure 32. DSC curves of iCOFMs (TpBd-SO_3H and TpPa-SO_3H)

Note: Before DSC test, TpBd-SO_3H and TpPa-SO_3H were pretreated at 100 °C for 24 h to remove the free water. The DSC results are shown in Fig. 32. No endothermic peak is observed at around 0°C owing to the absence of ice or ice-like water. Both iCOFMs display obvious endothermic peak at around 100°C due to the cleavage of hydrogen bond between -SO_3H group and bound water.

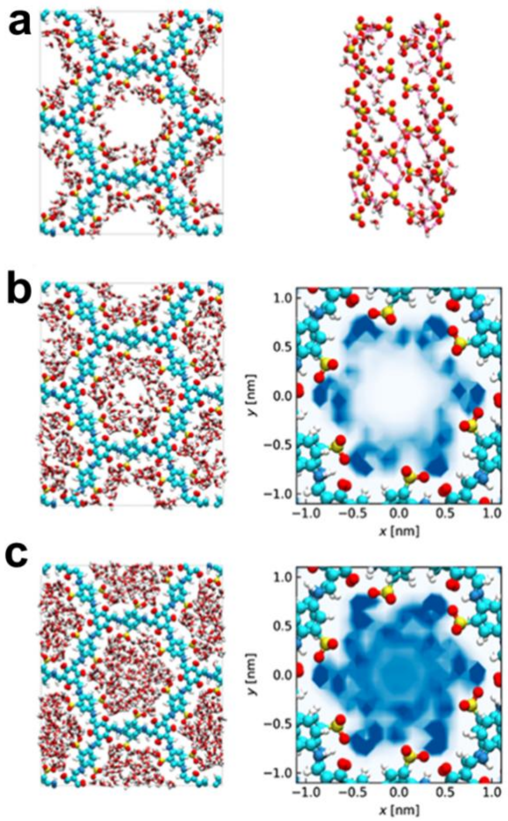

Supplementary Figure 33. Typical snapshots of (a) 300 H_2O plus 120 H_3O^+, (b) 600 H_2O plus 120 H_3O^+ and (c) 900 H_2O plus 120 H_3O^+ in ten stacked TpPa-SO_3H nanosheets. In (A), one snapshot to show the H-bond network (pink bonds) between H_2O and -SO_3H groups. In (B) and (C), the density profile of water molecules (plus the hydroniums) inside one nanochannel.

Note: The water aggregation state in iCOFM was investigated by molecular dynamics simulation. As shown in Fig.33, water molecules are disorderly aggregated in the iCOF nanochannel. When there are a few numbers of water molecules in the system (e.g. 300 H_2O and 120 H_3O^+ in Fig. 33a), all water molecules are adsorbed into the nanochannels and form a thin water film on the wall of the nanochannels. Inside the channels, we can see that -SO_3H groups are connected by water molecules and H-bonds. As we increase the number of H_2O, the water film grows and the nanochannels are gradually filled. Meanwhile, we can see the coalescence of the hydration shells around -SO_3H in the liquid water (e.g. 600 H_2O and 120 H_3O^+ in Fig. 33b, 900 H_2O and 120 H_3O^+ in Fig. 33c).

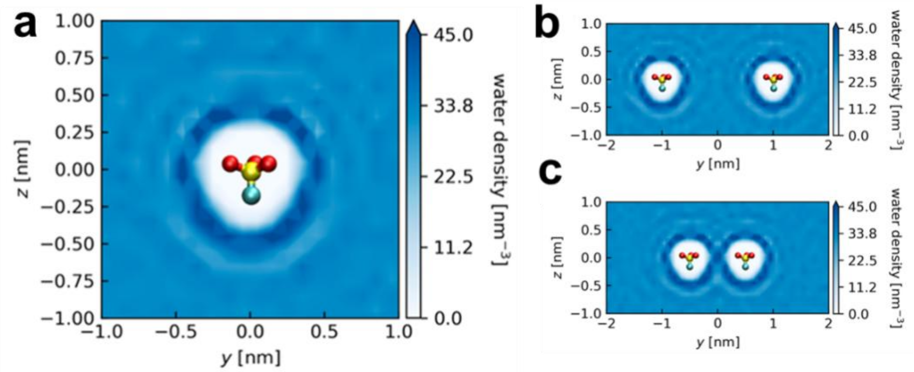

Supplementary Figure 34. (a) The density profile of water molecules around an isolated -SO_3H group. (b and c) The density profile of water molecules around two -SO_3H groups. O atoms are red, S atoms are yellow and C atoms are blue.

Note: Fig. 34 shows the density distribution of water molecules around -SO_3H group. Due to the strong molecular affinity between H_2O and -SO_3H, water molecule forms a significant adsorption layer (i.e. hydration shell) near the -SO_3H group, whose outer edge is away from the central S atom by ~0.5 nm (Fig. 34a). When there are two -SO_3H groups in the system, the structure of the water adsorption layers around them depends on the group distance. As shown in Fig. 34b, when the -SO_3H group distance is 2 nm, the adsorption layers are almost isolated, similar to that around an isolated -SO_3H group. However, when the group distance decrease to 1 nm, the two adsorption layers starts to coalesce, forming a dense water bridge between two hydration shells (Fig. 34c).

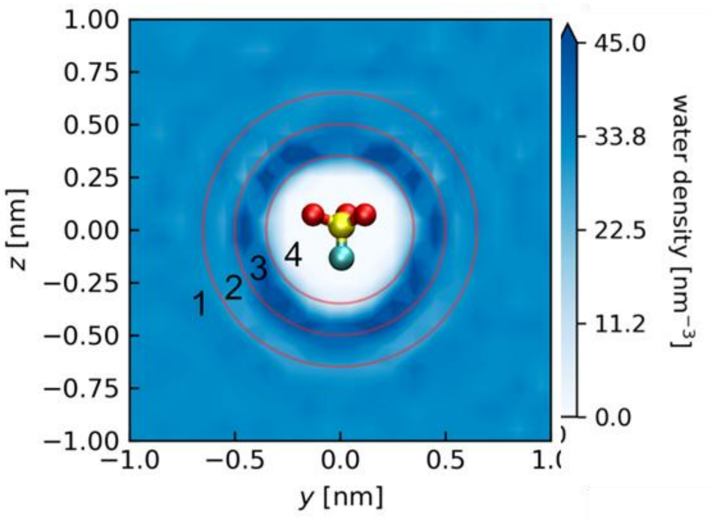

Supplementary Figure 35. The water molecules and hydrogen bond density around an isolated -SO_3H group.

Note: We analyzed the hydrogen bonds and water dynamics in the region 1-4. The boundary between these regions is at r=0.35, 0.5 and 0.65 nm. The calculated results show that, in average, each water in region 1-4 has close hydrogen bond number of 2.50, 2.50, 2.47, 2.47, respectively. According to water molecules density distribution, the hydration shell (region 3) has a higher hydrogen bond density. Meanwhile, the water molecules in the hydration shell are not bonded to the -SO_3H group. By tracking water molecules in the hydration shell, we can see that these water molecules have frequent exchanges with bulk water molecules.

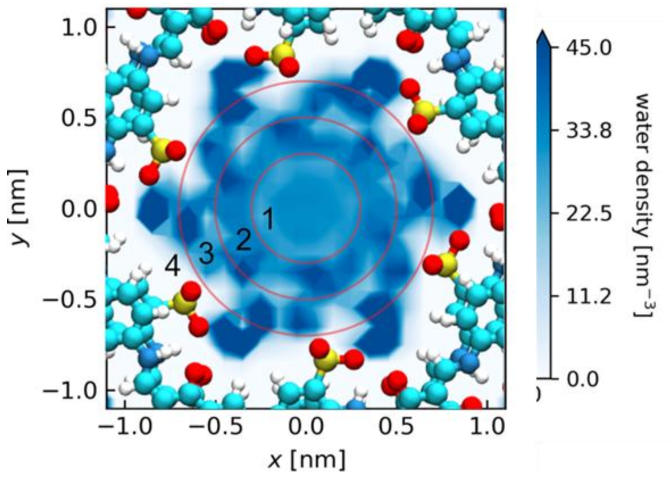

Supplementary Figure 36. The water molecules density inside one nanochannel in the system of 900 H_2O plus 120 H_3O^+.

Note: We analyzed the hydrogen bonds and water dynamics in iCOF in the region 1-4. The boundary between these regions is at r=0.3, 0.5 and 0.7 nm. The calculated results show that, in average, each water in region 1-4 has close hydrogen bond number of about 2.46. According to water molecules density distribution, the hydration shell (region 3) has a higher hydrogen bond density. Meanwhile, the water molecules in the hydration shell are not bonded to the -SO_3H group.

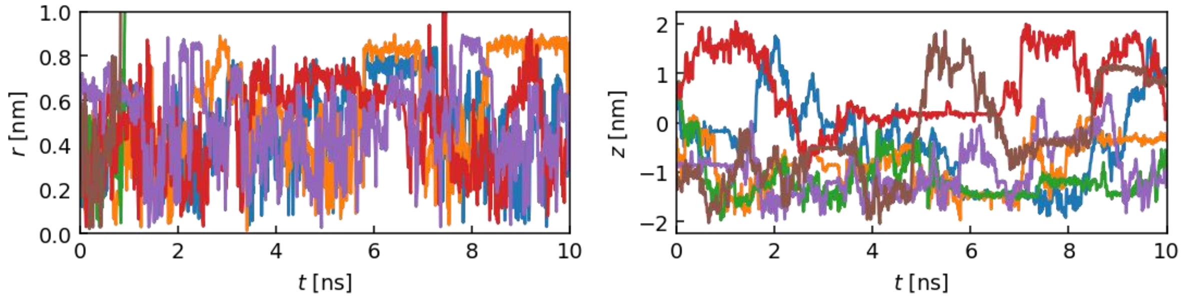

Supplementary Figure 37. The radial position (r) and longitudinal axis position (z) of some water molecules in the hydration shell. The horizontal fluctuant curves represent the periods of water being trapped inside the channel.

Note: For water molecules in the hydration shell, they can frequently exchange with water molecules in other regions. However, some water molecules can be trapped between two -SO_3H groups (or two COF layers) for a while. As a result, the transport of water molecules along the longitudinal axis inside the channel can be slown down.

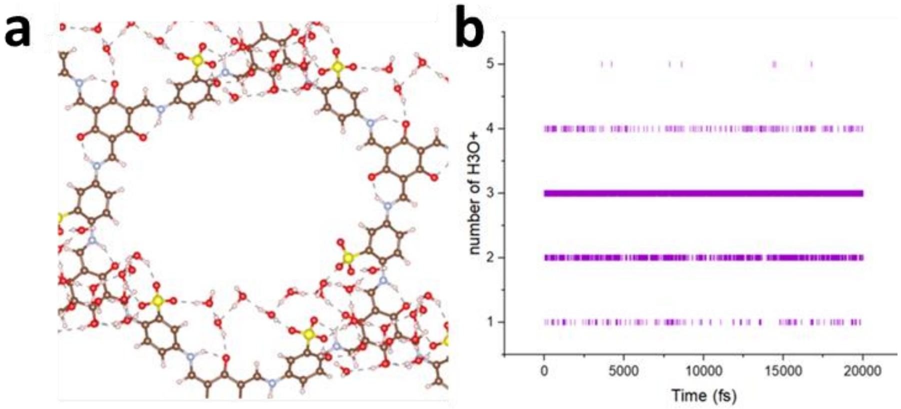

Supplementary Figure 38. AIMD simulation results for TpPa-SO_3H. (a) The configuration of TpPa-SO_3H with hydration number of 7. (b) The evolution of the number of the H_3O^+ ions over a time period of 20000 fs.

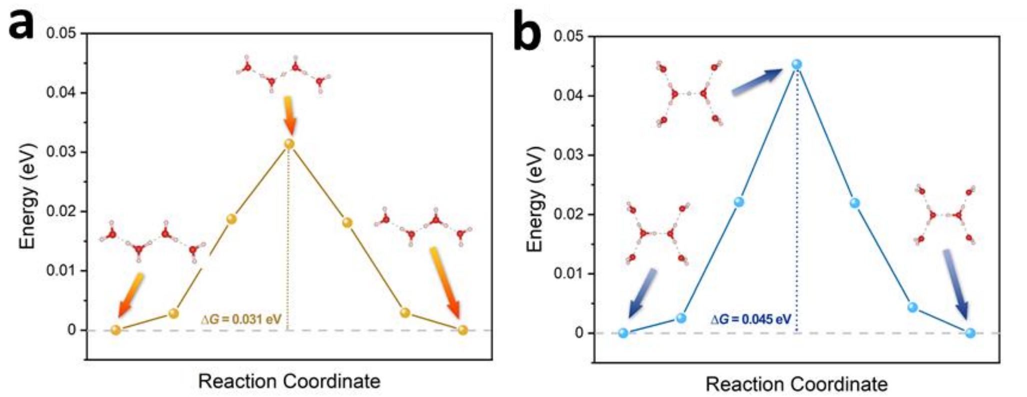

Supplementary Figure 39. Grotthuss diffusion of proton in a sequence of water molecules. (a) Linear configuration, in which each water molecule forms two hydrogen bonds with its neighbors. (b) Planar configuration, in which each water molecule forms three hydrogen bonds with its neighbors.

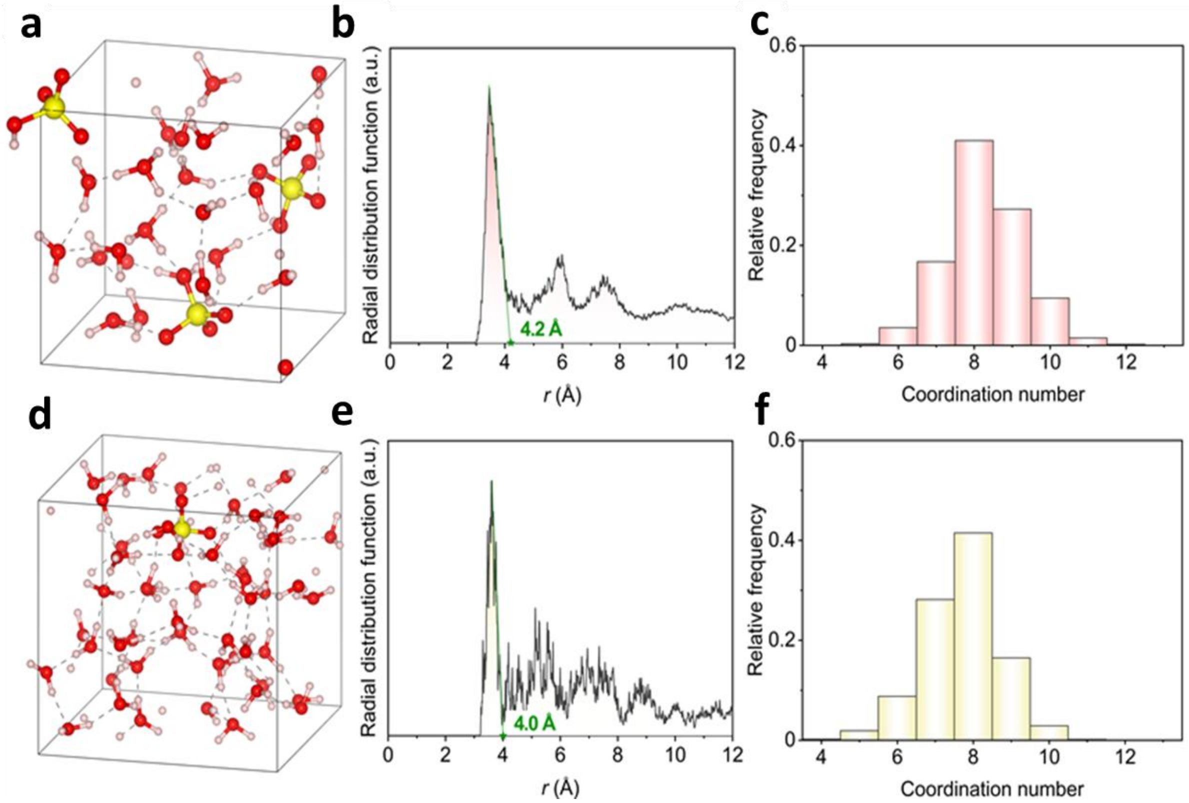

Supplementary Figure 40. Coordination of SO_4^+ ions in H_2SO_4 solution. a,d, Structures of H_2SO_4 solutions with H_2SO_4:H_2O = 1:7 (a) and H_2SO_4:H_2O = 1:50 (d). b,e, Radial distribution functions of H_3O^+ (coordinates represented by the O atom) with respect to the SO_4^+ ions (coordinates represented by S) in H_2SO_4 solutions with H_2SO_4:H_2O = 1:7 (b) and H_2SO_4:H_2O = 1:50 (e). c, f, Distribution of the coordination number of H_2O with respect to SO_4^+ in solutions with H_2SO_4:H_2O = 1:7 (c) and H_2SO_4:H_2O = 1:50 (f). The distance thresholds for defining the nearest-neighboring H_2O molecules are 4.2 and 4.0 Å for H_2SO_4:H_2O = 1:7 and H_2SO_4:H_2O = 1:50, respectively.

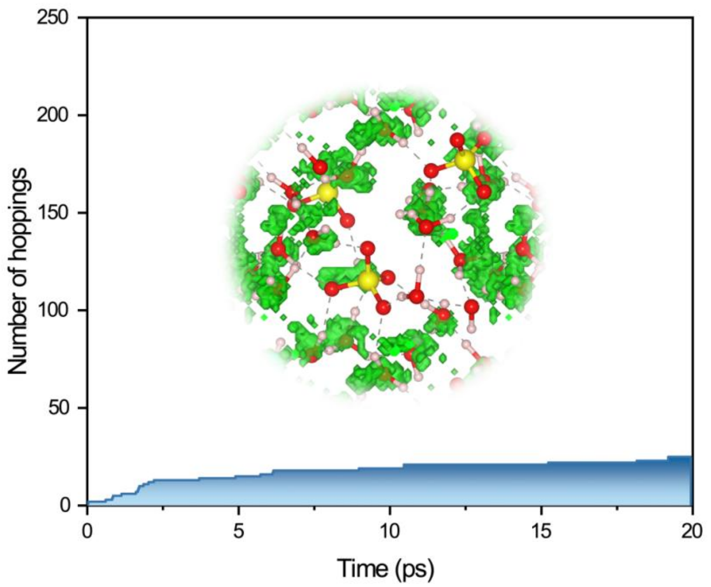

Supplementary Figure 41. Number of proton transport events in H_2SO_4 solution. Simulation is performed on a H_2SO_4 solution with H_2SO_4:H_2O = 1:7. The probability density of H_3O^+ (coordinates represented by the O atom) is shown in the inset.

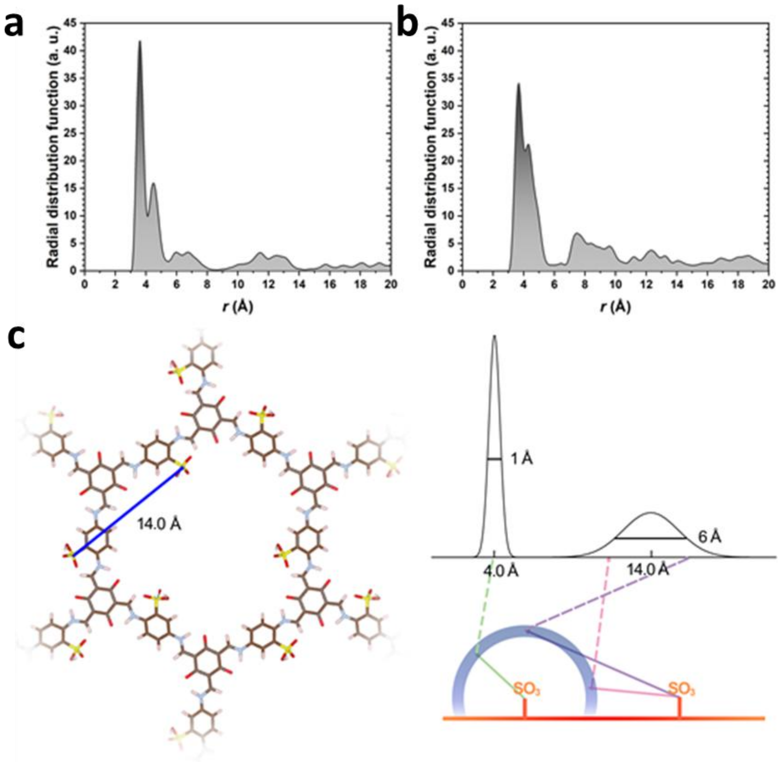

Supplementary Figure 42. Distance of H_3O^+ to sulfonate ligand. a,b, Radial distribution functions of H_3O+ (coordinates represented by the O atom) when taking S atoms in TpPa-SO_3H (a) and TpBd-SO_3H (b) as the reference centers. (c) Schematic illustration of the smearing of probability density distribution of H_3O^+ in TpPa-SO_3H.

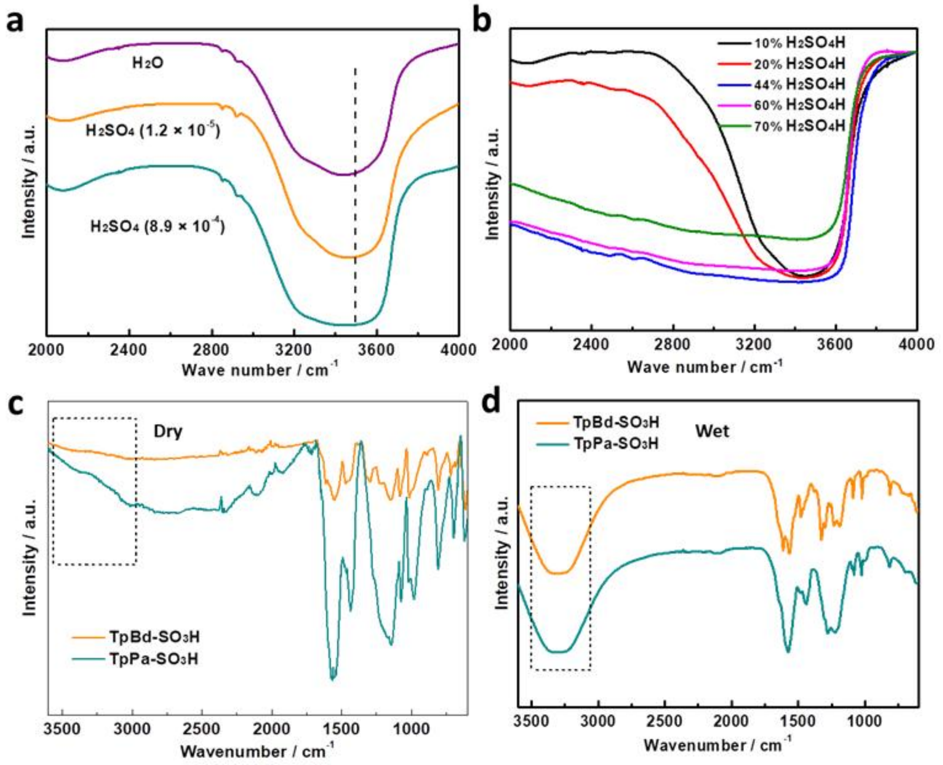

Supplementary Figure 43. FTIR spectrum of water, H_2SO_4 solution (a and b), iCOFM (c(dry) and d (wet)).

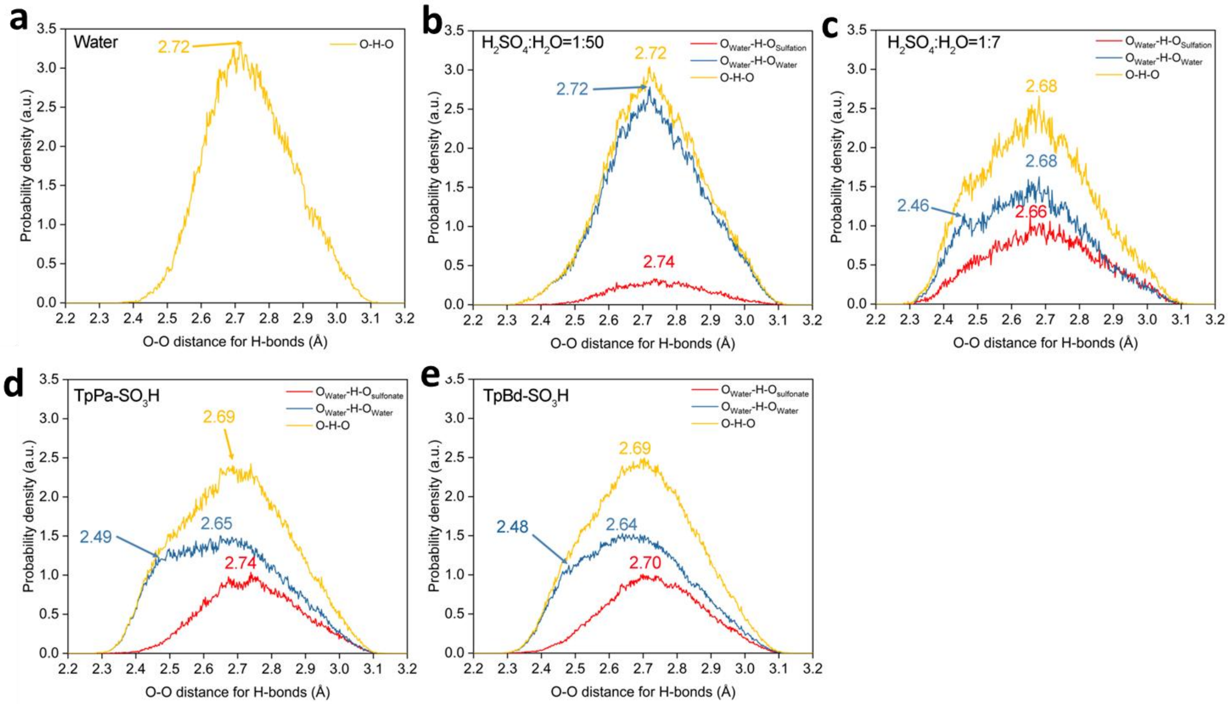

Supplementary Figure 44. The O-O distance in water (a), H_2SO_4 solution (b and c), TpPa-SO_3H (d), and TpBd-SO_3H (e).

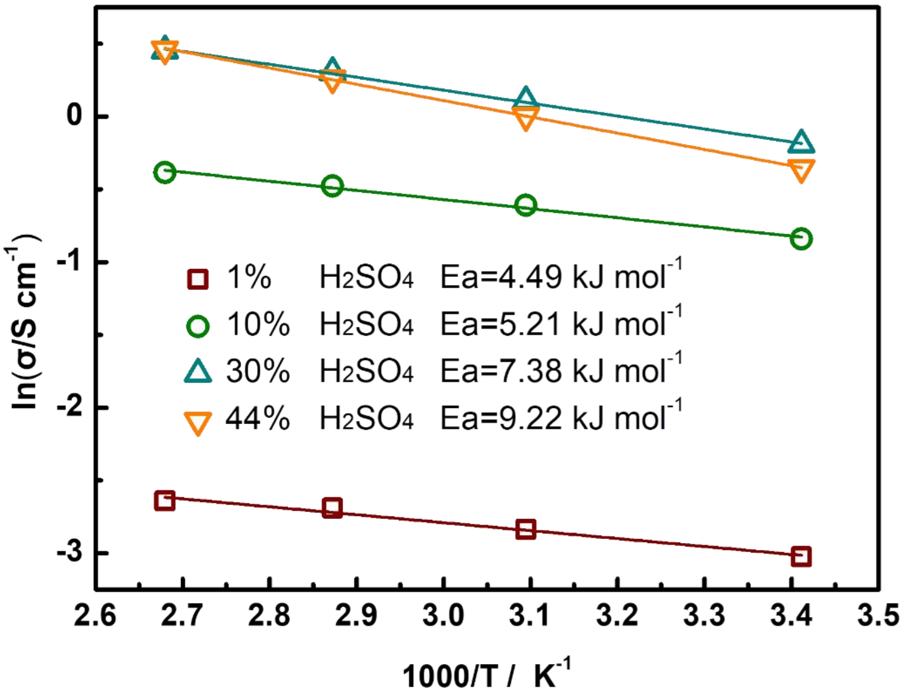

Supplementary Figure 45. Temperature dependent proton conductivity of H_2SO_4 solution.

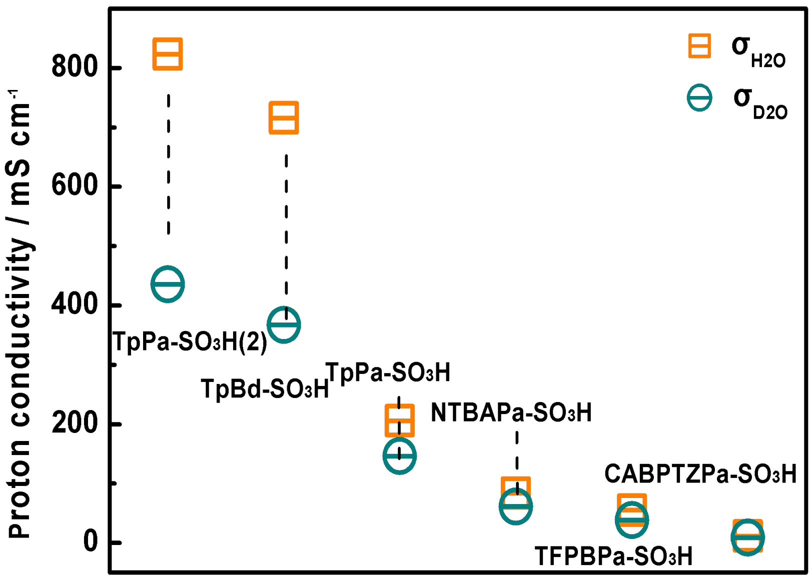

Supplementary Figure 46. Proton conductivity of iCOFM at 30 °C under H_2O or D_2O vapor.

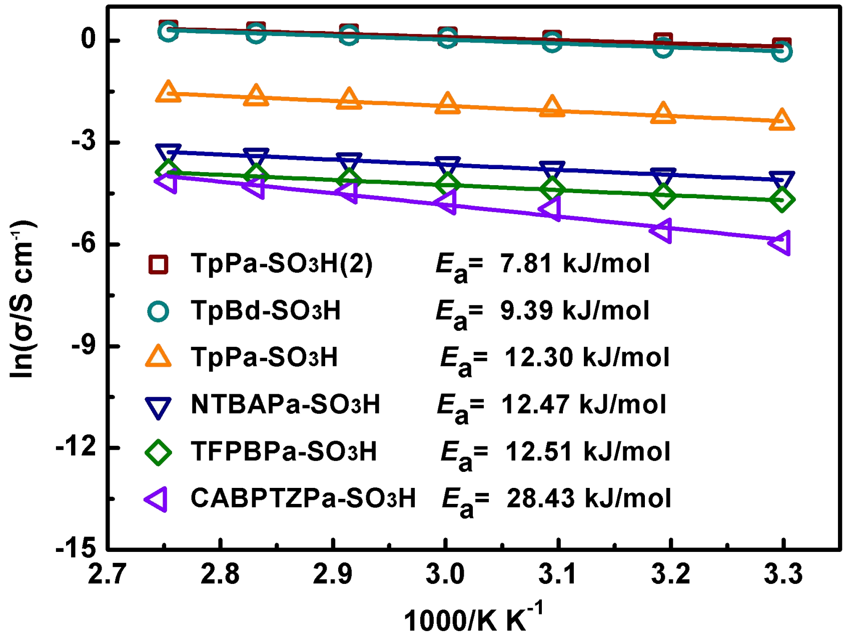

Supplementary Figure 47. Arrhenius plots of proton conductivities of iCOFMs.

References

1. Park, G. C. & Kim, D. Porous PTFE reinforced SPEEK proton exchange membranes for enhanced mechanical, dimensional, and electrochemical stability. *Polymer* **218**, 123506 (2021).
2. Sood, R. *et al.* Active electrospun nanofibers as an effective reinforcement for highly conducting and durable proton exchange membranes. *Journal of Membrane Science* **622**, (2021).
3. Paul, S., Choi, S. J. & Kim, H. J. Co-tri MOF-impregnated Aquivion® composites as proton exchange membranes for fuel cell applications. *Ionics* **27**, 1653–1666 (2021).
4. Raja, K., Raja Pugalenthi, M. & Ramesh Prabhu, M. Investigation on the sulfonated poly(ether ether ketone)/poly(amide-imide)/barium cerate-based nanocomposite membrane for proton exchange membrane fuel cells. *International Journal of Energy Research* **45**, 8564–8576 (2021).
5. Cai, Y. Y. *et al.* Bioinspired layered proton-exchange membranes with high strength and proton conductivity. *International Journal of Hydrogen Energy* **46**, 4087–4099 (2020).
6. Qian, L. *et al.* Magnetic aligned sulfonated carbon nanotube/Nafion composite membranes with anisotropic mechanical and proton conductive properties. *Journal of Materials Science* **56**, 6764–6779 (2021).
7. Chen, W. *et al.* SO42-/SnO2Solid Superacid Granular Stacked One-Dimensional Hollow Nanofiber for a Highly Conductive Proton-Exchange Membrane. *ACS Applied Materials and Interfaces* **12**, 40740–40748 (2020).
8. Wang, L. *et al.* Construction of Interpenetrating Transport Channels and Compatible Interfaces via a Zeolitic Imidazolate Framework 'bridge' for Nanofibrous Hybrid PEMs

with Enhanced Proton Conduction and Methanol Resistance. *ACS Sustainable Chemistry and Engineering* **8**, 12976–12989 (2020).

9. Guo, X., Fan, Y., Xu, J., Wang, L. & Zheng, J. Amino-MIL-53(Al)-nanosheets@Nafion composite membranes with improved proton/methanol selectivity for passive direct methanol fuel cells. *Industrial and Engineering Chemistry Research* **59**, 14825–14833 (2020).

10. Vinothkannan, M., Ramakrishnan, S., Kim, A. R., Lee, H. K. & Yoo, D. J. Ceria Stabilized by Titanium Carbide as a Sustainable Filler in the Nafion Matrix Improves the Mechanical Integrity, Electrochemical Durability, and Hydrogen Impermeability of Proton-Exchange Membrane Fuel Cells: Effects of the Filler Content. *ACS Applied Materials and Interfaces* **12**, 5704–5716 (2020).

11. Ghorai, A., Mandal, A. K. & Banerjee, S. Synthesis and Characterization of New Phosphorus Containing Sulfonated Polytriazoles for Proton Exchange Membrane Application. *Journal of Polymer Science* **58**, 263–279 (2020).

12. Han, D., Hossain, S. I., Son, B., Lee, D. H. & Shanmugam, S. Pyrochlore Zirconium Gadolinium Oxide Nanorods Composite Membrane for Suppressing the Formation of Free Radical in PEM Fuel Cell Operating under Dry Condition. *ACS Sustainable Chemistry and Engineering* **7**, 16889–16899 (2019).

13. Sui, Y., Du, Y., Hu, H., Qian, J. & Zhang, X. Do acid-base interactions really improve the ion conduction in a proton exchange membrane?-a study on the effect of basic groups. *Journal of Materials Chemistry A* **7**, 19820–19830 (2019).

14. Oh, K., Kwon, O., Son, B., Lee, D. H. & Shanmugam, S. Nafion-sulfonated silica composite membrane for proton exchange membrane fuel cells under operating low

humidity condition. *Journal of Membrane Science* **583**, 103–109 (2019).

15. Gao, S. *et al.* Highly sulfonated poly(ether ether ketone) grafted on graphene oxide as nanohybrid proton exchange membrane applied in fuel cells. *Electrochimica Acta* **283**, 428–437 (2018).

16. Sasmal, H. S. *et al.* Superprotonic Conductivity in Flexible Porous Covalent Organic Framework Membranes. *Angewandte Chemie - International Edition* **57**, 10894–10898 (2018).

17. Li, J. *et al.* Non-destructive modification on Nafion membrane via in-situ inserting of sheared graphene oxide for direct methanol fuel cell applications. *Electrochimica Acta* **282**, 362–368 (2018).

18. Donnadio, A. *et al.* Mixed Membrane Matrices Based on Nafion/UiO-66/SO3H-UiO-66 Nano-MOFs: Revealing the Effect of Crystal Size, Sulfonation, and Filler Loading on the Mechanical and Conductivity Properties. *ACS Applied Materials and Interfaces* **9**, 42239–42246 (2017).

19. Zhang, H. *et al.* Fuel cell performance of pendent methylphenyl sulfonated poly(ether ether ketone ketone)s. *Journal of Power Sources* **368**, 30–37 (2017).

20. Wang, G. & Guiver, M. D. Proton exchange membranes derived from sulfonated polybenzothiazoles containing naphthalene units. *Journal of Membrane Science* **542**, 159–167 (2017).

21. Miyake, J. *et al.* Design of flexible polyphenylene proton-conducting membrane for next-generation fuel cells. *Science Advances* **3**, (2017).

22. Sun, H., Tang, B. & Wu, P. Rational Design of S-UiO-66@GO Hybrid Nanosheets for Proton Exchange Membranes with Significantly Enhanced Transport Performance. *ACS*

Applied Materials and Interfaces **9**, 26077–26087 (2017).

23. Salarizadeh, P., Javanbakht, M. & Pourmahdian, S. Enhancing the performance of SPEEK polymer electrolyte membranes using functionalized TiO2 nanoparticles with proton hopping sites. *RSC Advances* **7**, 8303–8313 (2017).

24. Li, J. *et al.* An in-situ nano-scale swelling-filling strategy to improve overall performance of Nafion membrane for direct methanol fuel cell application. *Journal of Power Sources* **332**, 37–41 (2016).

25. Salarizadeh, P., Javanbakht, M., Pourmahdian, S. & Beydaghi, H. Influence of amine-functionalized iron titanate as filler for improving conductivity and electrochemical properties of SPEEK nanocomposite membranes. *Chemical Engineering Journal* **299**, 320–331 (2016).

26. Ahmadian-Alam, L., Kheirmand, M. & Mahdavi, H. Preparation, characterization and properties of PVDF-g-PAMPS/PMMA-co-PAMPS/silica nanoparticle as a new proton exchange nanocomposite membrane. *Chemical Engineering Journal* **284**, 1035–1048 (2016).

27. Liu, D. *et al.* Synthesis and properties of highly branched sulfonated poly(arylene ether)s with flexible alkylsulfonated side chains as proton exchange membranes. *Journal of Materials Chemistry C* **4**, 1326–1335 (2016).

28. Liu, K. L. *et al.* Sulfonated poly(styrene-block-(ethylene-ran-butylene)-block-styrene (SSEBS)-zirconium phosphate (ZrP) composite membranes for direct methanol fuel cells. *Journal of Membrane Science* **495**, 110–120 (2015).

29. Pang, J. *et al.* Fluorinated poly(arylene ether ketone) containing pendent hexasulfophenyl for proton exchange membrane. *Journal of Membrane Science* **492**, 67–76 (2015).

30. Wu, Z. *et al.* Linear sulfonated polyimides containing polyhedral oligomeric silsesquioxane (POSS) in main chain for proton exchange membranes. *Journal of Power Sources* **290**, 42–52 (2015).

31. Ko, T. *et al.* Cross-linked sulfonated poly(arylene ether sulfone) membranes formed by in situ casting and click reaction for applications in fuel cells. *Macromolecules* **48**, 1104–1114 (2015).

32. Hamada, T. *et al.* Poly(ether ether ketone) (PEEK)-based graft-type polymer electrolyte membranes having high crystallinity for high conducting and mechanical properties under various humidified conditions. *Journal of Materials Chemistry A* **3**, 20983–20991 (2015).

33. Ko, T. *et al.* Sulfonated poly(arylene ether sulfone) composite membranes having poly(2,5-benzimidazole)-grafted graphene oxide for fuel cell applications. *Journal of Materials Chemistry A* **3**, 20595–20606 (2015).

34. Cui, M. *et al.* Proton-conducting membranes based on side-chain-type sulfonated poly(ether ketone/ether benzimidazole)s via one-pot condensation. *Journal of Membrane Science* **465**, 100–106 (2014).

35. Cheng, H. *et al.* Preparation and characterization of sulfonated poly(arylene ether ketone) copolymers with pendant sulfoalkyl groups as proton exchange membranes. *Journal of Power Sources* **260**, 307–316 (2014).

36. Wen, P. *et al.* A novel approach to prepare photocrosslinked sulfonated poly(arylene ether sulfone) for proton exchange membrane. *Journal of Membrane Science* **463**, 58–64 (2014).

37. He, G. *et al.* Constructing facile proton-conduction pathway within sulfonated poly(ether ether ketone) membrane by incorporating poly(phosphonic acid)/silica nanotubes. *Journal of Power Sources* **259**, 203–212 (2014).

38. Yao, B. *et al.* Synthesis of sulfonic acid-containing polybenzoxazine for proton exchange membrane in direct methanol fuel cells. *Macromolecules* **47**, 1039–1045 (2014).

39. Yu, D. M. *et al.* Properties of sulfonated poly(arylene ether sulfone)/electrospun nonwoven polyacrylonitrile composite membrane for proton exchange membrane fuel cells. *Journal of Membrane Science* **446**, 212–219 (2013).

40. Zhang, L. *et al.* High proton-conducting polymer electrolytes based on pendent poly(arylene ether ketone) with H-bond for proton exchange membranes. *International Journal of Hydrogen Energy* **38**, 12363–12373 (2013).

41. Mukherjee, R., Mohanty, A. K., Banerjee, S., Komber, H. & Voit, B. Phthalimidine based fluorinated sulfonated poly(arylene ether sulfone)s copolymer proton exchange membranes. *Journal of Membrane Science* **435**, 145–154 (2013).

42. Feng, S., Shen, K., Wang, Y., Pang, J. & Jiang, Z. Concentrated sulfonated poly (ether sulfone)s as proton exchange membranes. *Journal of Power Sources* **224**, 42–49 (2013).

43. Wang, G., Yao, Y., Xiao, G. & Yan, D. Novel sulfonated polybenzothiazoles with outstanding dimensional stability for proton exchange membranes. *Journal of Membrane Science* **425–426**, 200–207 (2013).

44. Zhang, X. *et al.* Preparation and properties of novel sulfonated poly(p-phenylene-co-aryl ether ketone)s for polymer electrolyte fuel cell applications. *Journal of Power Sources* **216**, 261–268 (2012).

45. Enomoto, K., Takahashi, S., Rohani, R. & Maekawa, Y. Synthesis of copolymer grafts containing sulfoalkyl and hydrophilic groups in polymer electrolyte membranes. *Journal of Membrane Science* **415–416**, 36–41 (2012).

46. Wang, J. *et al.* Cross-linked proton exchange membranes for direct methanol fuel cells:

Effects of the cross-linker structure on the performances. *International Journal of Hydrogen Energy* **37**, 12586–12596 (2012).

47. Wang, C. *et al.* Poly(arylene ether sulfone) proton exchange membranes with flexible acid side chains. *Journal of Membrane Science* **405–406**, 68–78 (2012).

48. Kim, J. E. & Kim, D. Pendant-sulfonated poly(arylene ether ketone) (PAEK) membranes cross-linked with a proton conducting reagent for fuel cells. *Journal of Membrane Science* **405–406**, 176–184 (2012).

49. Cao, L. *et al.* Weakly Humidity-Dependent Proton-Conducting COF Membranes. *Advanced Materials* **32**, 1–9 (2020).

50. Qiu, M. *et al.* Metal-Organic Nanogel with Sulfonated Three-Dimensional Continuous Channels as a Proton Conductor. *ACS Applied Materials and Interfaces* **12**, 19788–19796 (2020).

51. Liu, Y. *et al.* Sulfonated lignin intercalated graphene oxide membranes for efficient proton conduction. *Journal of Membrane Science* **644**, 120126 (2022).
